# Optimization design of interdigitated microelectrodes with an insulation layer on the connection tracks to enhance efficiency of assessment of the cell viability

**DOI:** 10.1186/s42490-023-00070-w

**Published:** 2023-05-01

**Authors:** Sameh. Sherif, Yehya H. Ghallab, Omnia AbdelRaheem, Laila Ziko, Rania Siam, Yehea Ismail

**Affiliations:** 1 Biomedical Engineering Department, Helwan University, Cairo, Egypt; 2 Center of Nanoelectronics and Devices (CND), Zewail City of Science and Technology and The American University in Cairo (AUC), Cairo, Egypt; 3 Department of Biology, School of Sciences and Engineering, The American University in Cairo(AUC), Cairo, Egypt; 4 School of Life and Medical Sciences, the University of Hertfordshire, Hosted By Global Academic Foundation, Cairo, Egypt

**Keywords:** Impedance, Microelectrical Impedance Spectroscopy (µEIS), Microfluidic, Interdigitated Microelectrodes, MCF7, Microbeads

## Abstract

**Background:**

Microelectrical Impedance Spectroscopy (µEIS) is a tiny device that utilizes fluid as a working medium in combination with biological cells to extract various electrical parameters. Dielectric parameters of biological cells are essential parameters that can be extracted using µEIS. µEIS has many advantages, such as portability, disposable sensors, and high-precision results.

**Results:**

The paper compares different configurations of interdigitated microelectrodes with and without a passivation layer on the cell contact tracks. The influence of the number of electrodes on the enhancement of the extracted impedance for different types of cells was provided and discussed. Different types of cells are experimentally tested, such as viable and non-viable MCF7, along with different buffer solutions. This study confirms the importance of µEIS for in vivo and in vitro applications. An essential application of µEIS is to differentiate between the cells’ sizes based on the measured capacitance, which is indirectly related to the cells’ size. The extracted statistical values reveal the capability and sensitivity of the system to distinguish between two clusters of cells based on viability and size.

**Conclusion:**

A completely portable and easy-to-use system, including different sensor configurations, was designed, fabricated, and experimentally tested. The system was used to extract the dielectric parameters of the Microbeads and MCF7 cells immersed in different buffer solutions. The high sensitivity of the readout circuit, which enables it to extract the difference between the viable and non-viable cells, was provided and discussed. The proposed system can extract and differentiate between different types of cells based on cells’ sizes; two other polystyrene microbeads with different sizes are tested. Contamination that may happen was avoided using a Microfluidic chamber. The study shows a good match between the experiment and simulation results. The study also shows the optimum number of interdigitated electrodes that can be used to extract the variation in the dielectric parameters of the cells without leakage current or parasitic capacitance.

**Supplementary Information:**

The online version contains supplementary material available at 10.1186/s42490-023-00070-w.

## Introduction

Breast cancer is the second leading cause of cancer-related deaths among women [1]. It originates in the epithelial cells of the breast tissue’s terminal ductal lobular [2] system. It is categorized and described using various techniques, such as pathology-based approaches based on the morphological characteristics of tumors [3–6]. However, many breast cancers (around 40–75%) cannot be adequately classified [5, 7]. Another significant concern about this cancer is reducing its high mortality rate globally. Hence, researchers are developing more efficient examination and identification techniques for cancer cells, which could benefit drug delivery [8, 9]. The analysis of the main features of breast cancer is the primary focus of different studies. Conventional methods for diagnosing breast cancer, such as pathological tissue analysis, surgery, and radiography [10], have drawbacks. For instance, microscopic evaluation may be necessary for a sample’s diagnosis, which can be time-consuming and preparatory.

Moreover, this process can be associated with a risk of false positives for many diagnostic and drug therapy applications [10]. Conventional techniques such as Fluorescence Activated Cell Sorting (FACS) and Magnetic Activated Cell Sorting (MACS) [11], which rely on specific fluorescent or magnetic markers attached to the surface of the tested cells, have their drawbacks as well. These markers can affect the dielectric parameters of the cell, and the accuracy of the results obtained [11]. Markers can also harm cell metabolism, making the cells unable to grow and divide properly [12]. The drawbacks of Marker usage can be bypassed by non-invasive and marker-free techniques [12].

Furthermore, conventional techniques are often complex, costly, and time-consuming, and the labeling step can significantly alter the final analyzed sample, thus limiting their use in various analyses [13]. The drawbacks of these conventional techniques can be avoided by using micro-electrical impedance spectroscopy (µEIS) [14], a non-invasive procedure used to analyze the electrical properties of cells or tissues. It involves applying an alternating current (AC) signal to the sample and measuring the resulting impedance, i.e., measuring the resistance of the sample to the flow of current. The impedance of a sample depends on its electrical properties, such as electrical conductivity and capacitance, and it can provide information about the structure and function of the sample. µEIS has many advantages over other techniques for analyzing the electrical properties of cells. It is non-invasive, meaning it doesn’t require the insertion of electrodes or other probes into the sample. It is also label-free, meaning it doesn’t need markers or labels that may affect the viability or behavior of the cells. µEIS can be used to analyze the electrical properties of cells in real time [15], which makes it useful for studying dynamic processes, such as cell proliferation and death [14, 16–18]. Various applications of µEIS [19], such as cell growth detection [20, 21], demonstrate that impedance spectroscopy allows for cell growth on the electrodes and the extraction of the impedance variation while the cell grows. This selected technique had a high response time due to its attachment strategy. Also, [21] presented a microfluidic setup for HeLa cell impedance detection and its corresponding circuit model.µEIS has been widely used to study various biological systems, including cancer cells. It has the potential for use in the diagnosis and treatment of cancer. To increase cell viability, [17] presents a method for delivering chemicals into specific cells. The technique utilized impedance measurements to enhance cell viability and efficiency during the recovery phases of individual cells post-electroporation [17, 18, 22–24]. µEIS has also been utilized to investigate the electrical properties of tissues such as brain tissue and to develop biosensors for detecting specific molecules in biological samples. The passive electrical properties and dielectric characteristics [21], such as the impedance of biological cells, including human cancer cells, can be elucidated using µEIS, which can describe the fluctuation in cell impedance across a range of frequencies [25]. These fluctuations are due to the permittivity and conductivity contributions of various polarization processes that occur upon changes in the externally applied field [26]. Thus, defining the parameters, such as applied voltage and frequency, are essential for analysis to improve the system’s reliability in extracting cell dielectric features [21, 27, 28], including cancer, stem, and neural cells [25].µEIS has been used to study the properties of tissues and organelles, including changes in their composition or structure [29]. Researchers [30] also performed an impedance analysis on human cervical cancer cell lines as a function of frequency. At low frequencies, the cell membrane acts as an insulator, while at high frequencies, it behaves as a conductor. In other words, the measured electrical impedance at low frequencies only reflects the impedance of the extracellular fluid since the electric field passes around the cell membrane [25]. In contrast, at high frequencies, the cell membrane impedance is so tiny that it cannot block the incident electric field, causing the cell to become permeable [31]. Thus, the fluctuations in the electrical impedance of biological cells depend on the electric field penetrating the cell membrane and overcoming the impedance difference. Therefore, defining cell behavior based on identifying the electric field and the frequency range rather than at a specific frequency is critical. µEIS relies on applying an AC voltage signal to a tested sample and measuring the fluctuation in current. Different sample components can be selected and analyzed by varying the signal frequency, enabling researchers to determine the dielectric features of the tested cells, such as conductivity and permittivity, and consequently, define their composition and structure. By analyzing the dielectric properties of cells and tissues, µEIS can be used to diagnose various diseases [32–34]. Another promising application that can be used with µEIS is to monitor the response and behavior of bacterial cells, evaluate different treatment techniques, and analyze the metabolic activity of the cells [35], as described in [33, 36]. µEIS system depends on various key factors, including the dielectric parameters of the cell, the suspending buffer at the electrode–electrolyte interface, and the sequence and arrangement of the sensing electrodes [37]. The tested cell’s permittivity and conductivity variations with frequency directly affect the impedance-extracted results [38, 39]. The extracted impedance of biological cells can be employed in various contexts, such as indicating cell status, attaching the cell, adhesion, or spreading [40]. Furthermore, leveraging the extracted impedance, biological particles and cells can be characterized as a fundamental electrical circuit comprising cytoplasmic resistance and membrane capacitance [41].

The shape of the electrodes used in µEIS is a crucial factor to consider. Various studies have shown that the electrodes’ shape significantly affects the accuracy and reproducibility of impedance measurements [42]. Multiple publications have evaluated different electrode constructions [43–46]. In general, the shape of the electrodes used in µEIS has significantly impacted the accuracy and reproducibility of measurements. Further research is required to optimize the electrode shape for improved performance. Other applications have been studied, such as using µEIS to analyze the extracted impedance signal from normal and cancerous red blood cells using 3D electrodes [47]. Additionally, a µEIS system employing a transparent multidisc electrode array has been proposed to evaluate the effectiveness of chitosan in reducing the toxicity of the breast cancer cell line MCF-7 [48].

Another critical factor that can influence the extracted impedance is the trajectory of the cells [49, 50]. It is crucial to prevent the divergence of the cells over the sensing electrodes. This factor has been addressed in previous studies, which utilized interdigitated microelectrodes instead of bipolar planar electrodes [25]. Recent studies have also shown that the µEIS system is affected by the electrode material, the geometrical arrangement of the electrodes, and the medium composition [39, 51, 52]. Additionally, changes in the temperature of the medium or buffer can also affect the µEIS system’s results [53, 54]. Therefore, it is essential to consider these factors when designing a µEIS system to obtain reliable results.

Different techniques have been used with µEIS for cell characterization, such as the hydrodynamics system [55], the negative dielectrophoresis technique for cell trapping [56, 57], and the dielectrophoresis force [58]. The effect of the double layer has also been improved using silver electrodes [59–61]. This study modified the system with a copper-free HAL fabrication and a silver coating to avoid toxic effects on biological cells. Similarly to [62], conventional impedance sensing is used. However, this study relies on a portable system with a Saleae logic analyzer as a control unit between the sensor and the GUI graphical user interface.

This study aims to improve the response time and the accuracy of µEIS. These improvements will be obtained by increasing the detection rate using an array of electrodes, decreasing the area of the electrodes, and avoiding the influence of cell position. However, the positional effect on the output signal amplitude presents a significant challenge to the precision and accuracy of impedance-based technology. Impedance measurements are affected by intrinsic particle properties and the particle trajectory through the sensing region. As a result, impedance signals obtained from identical particles passing through a microchannel can vary if they traverse the sensing zone along different paths.

For enhancing the accuracy of impedance extraction, technical improvements have been proposed. These improvements include: 1) control cell position within the microfluidic channel, 2) compensate for positional dependence through signal analysis, and 3) attempt to mitigate it based on specific electrode configuration or channel design. This research involves a simulation and experimental study using a micro-interdigitated approach based on the advantages of interdigitated electrodes compared to conventional bipolar electrodes [25]. Bipolar electrodes have several drawbacks, as outlined in [25], that prevent them from differentiating between cells based on the divergence over electrodes, reflected in the extracted impedance described in [25]. The basic impedance measurement technique relies on two electrodes, with an AC signal applied to one electrode and fluctuations in the signal from the other electrode being monitored. The change in the sensing signal predominantly depends on the dielectric properties of the cell positioned between the electrodes.

## Theory of operation

**Fig. 1:**
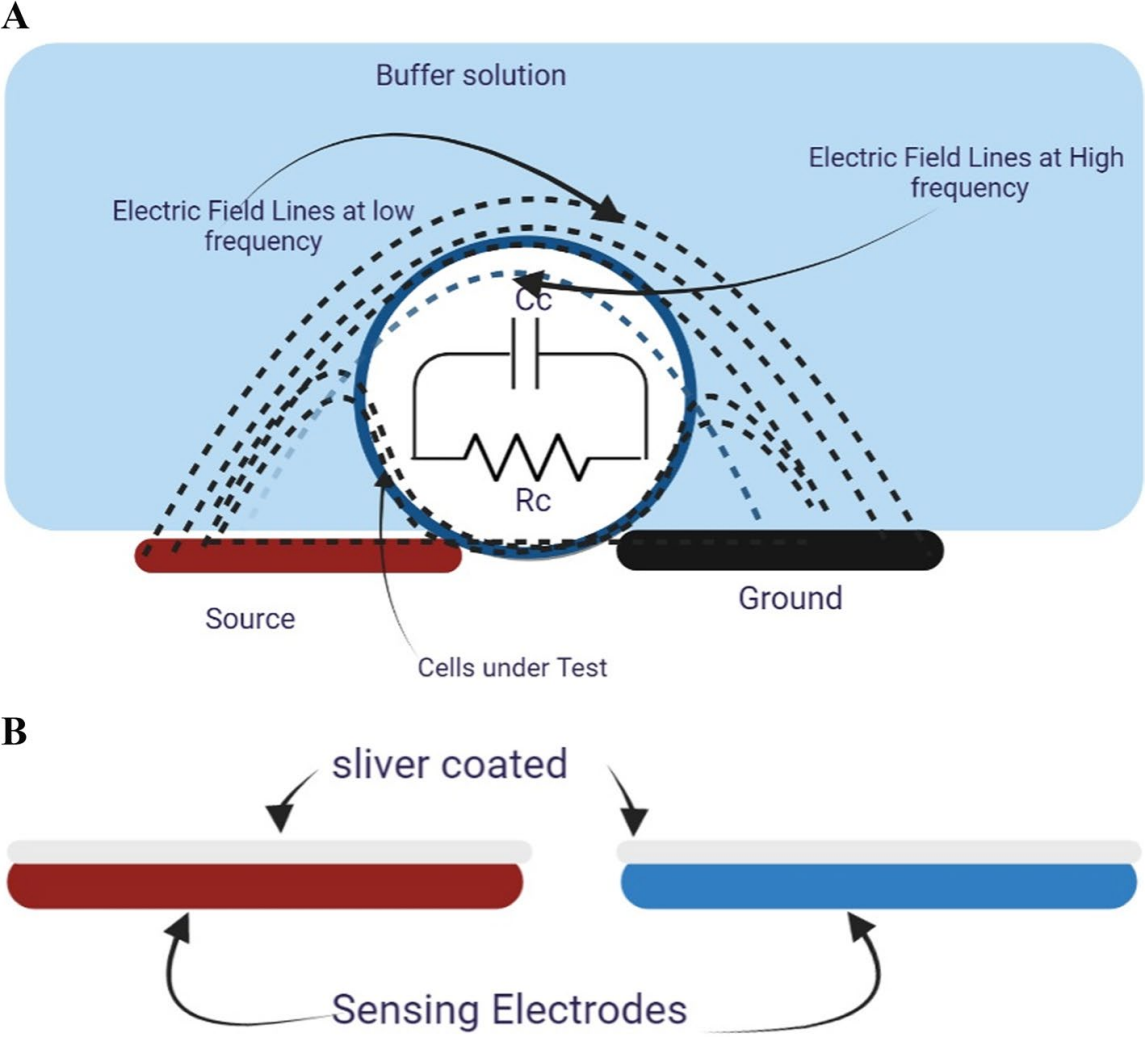
**A** shows the schematic single-cell model between coplanar electrodes and the equivalent circuit model utilized to determine the cells’ electrical characteristics and the electric field’s distribution. **B** The sensing electrodes are coated with silver to lessen the harmful effects of copper electrodes on the tested cell

The mathematical model used to define the biological cell using the sensing signal from the sensor electrode (Fig. 1A) is applied to synthetic and natural samples. Developing electrochemical sensors and biosensors involves three key steps: preparation, characterization methods, and testing. One crucial step in this process is electrode construction, which can be subject to the silver coating technique to avoid interference between copper and the tested cells (Fig. 1B). This technique provides insight into the ion channels. The electrical impedance is determined by the ratio of the applied voltage, E(jw), to the sensed current, A(jw), as a function of frequency in the domain, as depicted in Eq. (1).

1 $$The extracted Impedance =\frac{Applied Voltage}{Sensing Current}$$

2 $$The impedance Component=The real Component+The Imaginary Component$$

As stated in Eq. (1), the extracted impedance of a system is equal to the ratio of the applied voltage to the sensing current. Equation (2) indicates that the impedance of a system is composed of both a real and an imaginary component. These two equations describe the relationship between applied voltage, sensing current, and impedance.

When the cell is inserted between the sensing electrodes, as depicted in Fig. 1A, the cell-substrate impedance, Zc (Eq. 3), can be considered a combination of two primary passive elements (Fig. 1B). The electrical circuit [25] can be represented by the cell membrane capacitance, Cc, [related to the imaginary component], and the cell cytoplasm impedance [associated with the real part], which is defined by Rc and Rs referring to the buffer solution impedance. The extracted values of the cell impedance allow us to acquire electrophysiological and dielectric information about the cell.

3 $${Z}_{c}=\frac{1}{{\frac{1}{{R}_{c}}+}_{(jw{C}_{c})}}$$

## Signal generation system

**Fig. 2:**
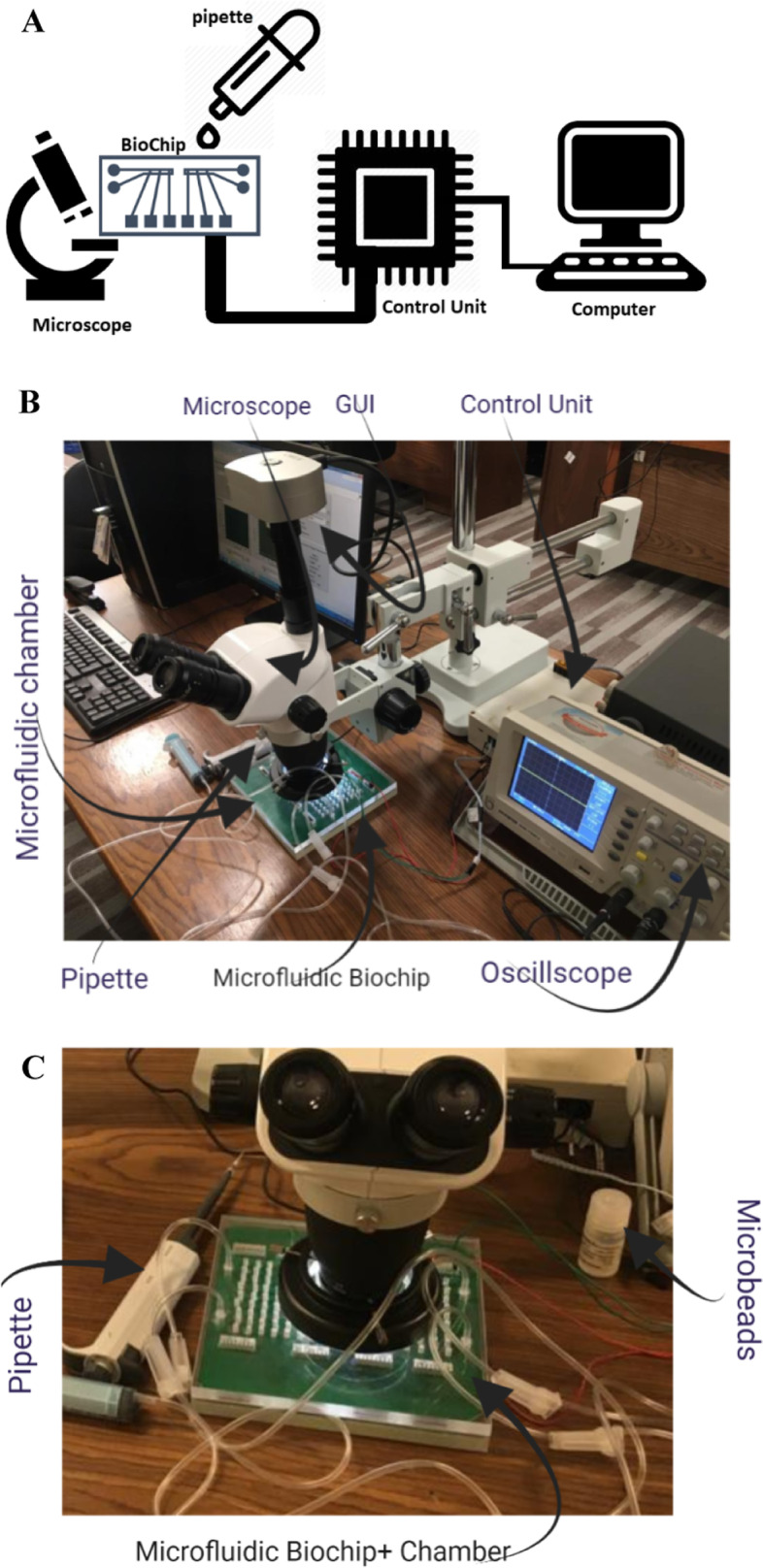
**A** a block diagram illustrates the systems utilized for extracting the cell’s impedance. Additionally, **B** depicts the system employed in the experimental study for extracting the impedance. Furthermore, **C** illustrates the microfluidic chamber with disposable decontamination techniques

The goal of this study is to utilize a portable system that has an easy-to-use interface. The control system and its components are described in this section to achieve this. The control system is connected to an excitation signal source and detector. The user can use the graphical user interface (GUI) to communicate with the system and define the extracted features, such as the strength of the excitation signal, the amplitude of the received signal, the frequency bandwidth, and the phase shift between the excitation and the sensed signal. The obtained data was then recorded in an Excel sheet for analysis using MATLAB software. Additionally, the readout circuit interacted with the system before and after cell insertion. The received signal (Erec) depends on the dielectric parameters of the inserted cells and buffer solution between the sensing electrodes. This circuit consists of an inverting amplifier AD844 that boosts the sensitivity, amplifies the sensing signal with an appropriate bandwidth matching the targeted frequency range, and prevents distortion. The second part of the system is the excitation signal (Eexc), which is applied to specified electrodes before the readout circuit collects the signal from the remaining electrodes. The excitation circuit generates a sinusoidal signal with an amplitude voltage of 3Vpp. The excitation signal is achieved in two steps: the first is to create 1 V using a Digital Synthesizer AD9851 DDS, and the second is to use an amplifier to achieve 3Vpp. The DDS controls the applied signal’s amplitude, phase, and frequency. Lastly, the display system uses Saleae Logic 8 to capture both the (Eexc) and (Erec) signals. The capture system using Saleae Logic is considered data acquisition with a sampling frequency of 50MS/s, thus allowing for communication with a sinusoidal signal at a high frequency of up to 5 MHz without aliasing. The resolution of the capture system with Saleae is 4.88 mV with 12 bits per sample, as specified in [25]. This system can be represented using Fig. 2A. It includes sensing electrodes with varying geometries, a control unit comprising an Eexc source and Erec detector, a microscopic system to track the cell as a reference tool, a GUI on the computer, and a pipette for cell volume control. Additionally, Fig. 2B illustrates the actual system comprising an oscilloscope as a reference tool to monitor the performance of the proposed control unit. The control unit can be employed without the oscilloscope, eliminating bulky restrictions and reducing costs. Lastly, Fig. 2C shows the microfluidic chamber, which prevents contamination and facilitates easy cleaning of the sensing electrodes after the Experiment. The chamber’s design is well-suited for microscopic and human visual inspection during sample injection.

## Method

### Proposed micro-electrical impedance system

**Fig. 3:**
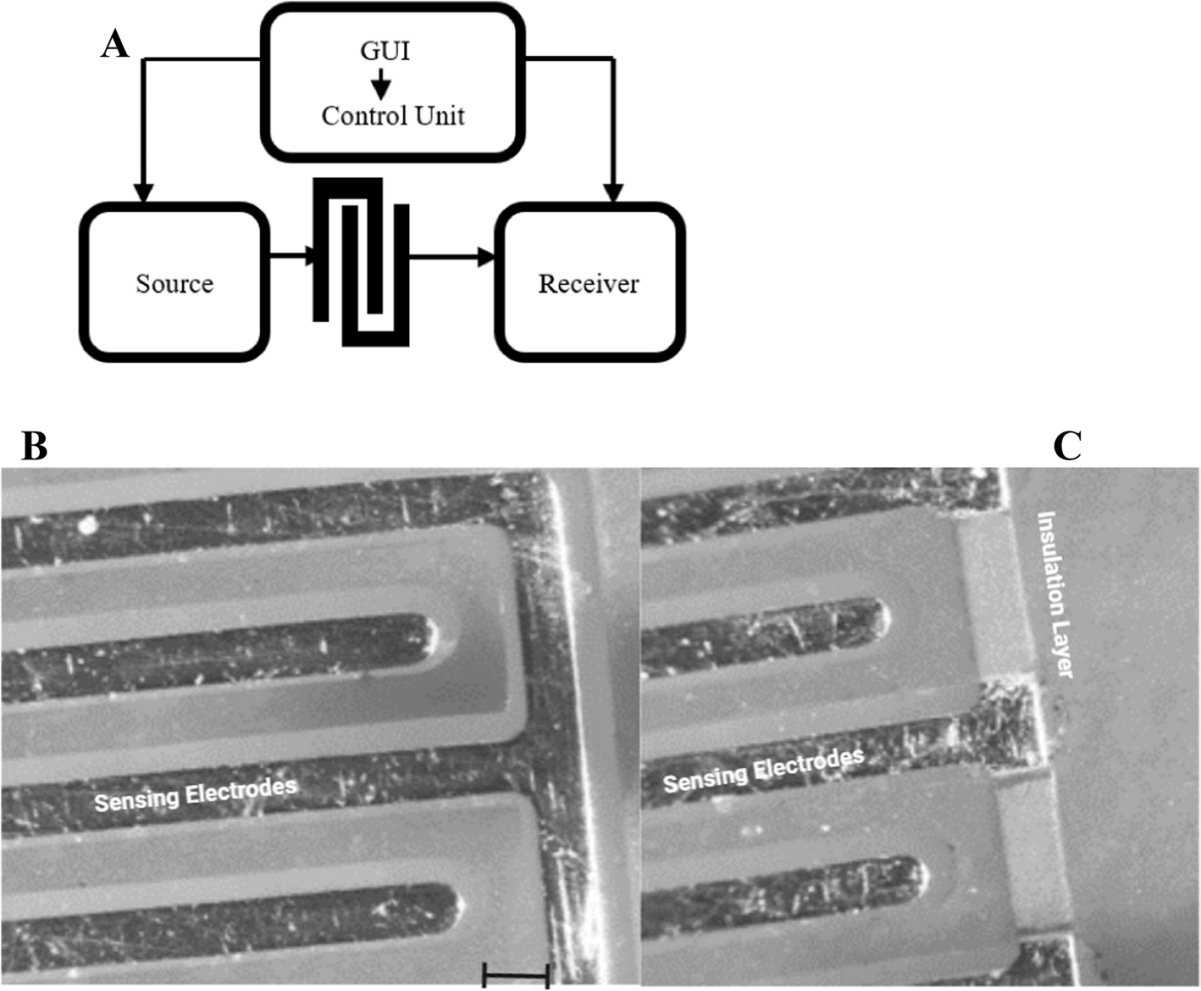
Illustrates the different components of the excitation, processing, and control units combined with interdigitated microelectrodes. Specifically, **A** presents a block diagram of the elements mentioned above. **B** and **C** provide microscopic images of interdigitated microelectrodes, with B depicting microelectrodes without insulation and C showing microelectrodes with an insulation layer

**Fig. 4:**
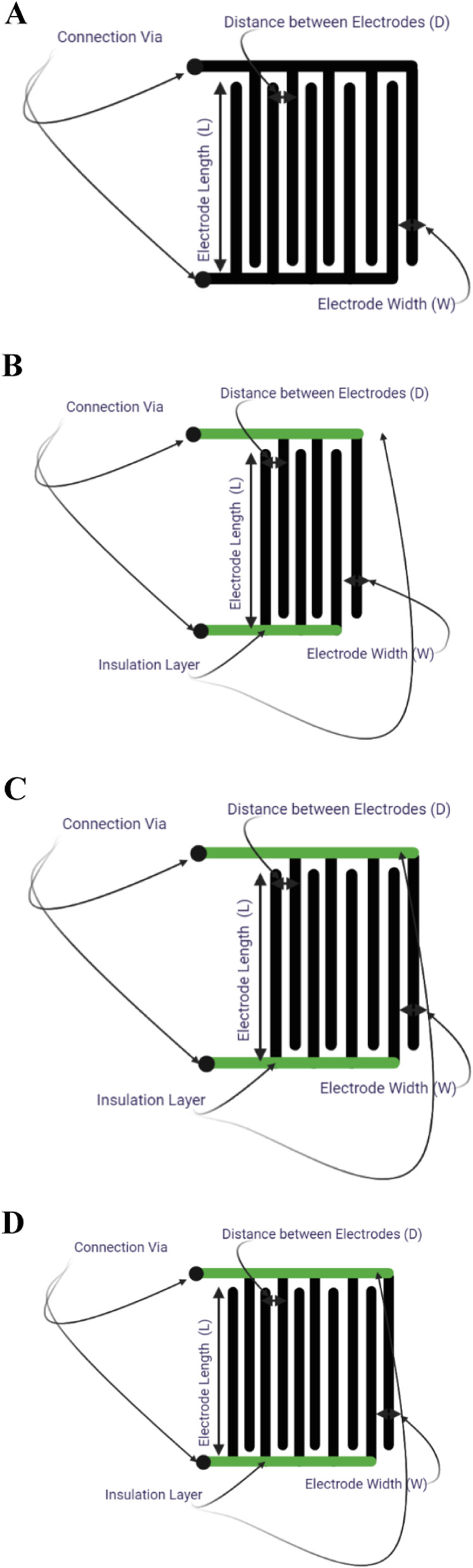
Illustrates four distinct interdigitated microelectrodes. **A** depicts the configuration of the interdigitated microelectrodes without an insulation layer, including the length, width, and distance between electrodes. **B** represents a different structure. **C** illustrates another configuration, and **D** shows the final configuration

The proposed micro-electrical impedance system aims to define the geometric description of the sensing electrodes, followed by the definition of the experimental steps and results, as depicted in Fig. 3A. The block diagram outlines the progressive steps for extracting impedance values. The connection between the excitation unit and the sensing electrodes is established to apply (Eexc), after which the sensing signal (Erec) is delivered to the processing unit. The interdigitated microelectrodes were fabricated using a cost-efficient 200-μm PCB technology and designed using ALTIUM Designer. The geometric parameters of the electrode configurations are as follows: length (L) = 6 mm, the distance between electrodes (D) = 250 µm, and thickness of electrodes (W) = 250 µm. The process’s sequence is managed using a control unit designed with an Arduino and connected to a Saleae logic analyzer for display purposes. Figure 3B shows a microscopic image of uninsulated interdigitated microelectrodes, while Fig. 3C shows the same with insulation. These figures demonstrate the main difference between the two main electrodes, which form the core of the Experiment before the number of electrodes was modified. The initial configuration selected is interdigitated microelectrodes (Fig. 4A) as the primary sensing electrode; due to the advantages outlined in [25], multiple electrodes can detect significant differences between the cells in the test based on those inserted between the measuring electrodes. Interdigitated electrodes also increase the detection rate, overcoming the solution’s divergence and avoiding cell trajectory. Another advantage of interdigitated microelectrodes compared to conventional bipolar microelectrodes is that they provide diagonal excitation and counteract the effect of cell trajectory. The number of electrodes in this configuration is 10, with no insulation layer on the connections, meaning all electrodes are included in the Experiment. The other designs use interdigitated microelectrodes with a coating or insulation layer (silkscreen) on the connection track to minimize leakage current and avoid additional capacitance. The analysis includes a comparison between different electrodes with varying numbers of electrodes, including the modification step of the insulation layer. Figure 4B shows the second selected design of interdigitated microelectrodes with an insulation layer; the number of electrodes in this design is six microelectrodes. The third configuration, shown in Fig. 4C, has eight electrodes, and the last configuration, shown in Fig. 4D, has ten microelectrodes. All these configurations of electrodes were presented and experimentally tested to extract the electrical parameters of MCF7 breast cancer cells and two types of polystyrene microbeads. A comparison between the experimental results of MCF7, both viable and non-viable cells, was presented and discussed. Both cells were tested when the buffer solution was PBS (Phosphate buffer saline) and DMEM Dulbecco’s modified Eagle’s medium. The concentration of the cells is described in Table S1.

The results of this study demonstrate the influence of various factors on impedance extraction, including the number of electrodes and the presence of an insulation layer. To further augment the research, simulation modeling using COMSOL is utilized to understand the electric field strength. The experimental results may guide researchers when conducting experiments with different cell sizes and assessing cell viability with high sensitivity. The frequency range of this study is between 10 kHz and 3 MHz, which encompasses Beta dispersion [63] and avoids high-frequency distortion. This range is chosen to consider the effects of both low and high frequencies; the low-frequency range allows for cell membrane polarization, while the high-frequency range penetrates the cell membrane [63].

## Control system and software

**Fig. 5:**
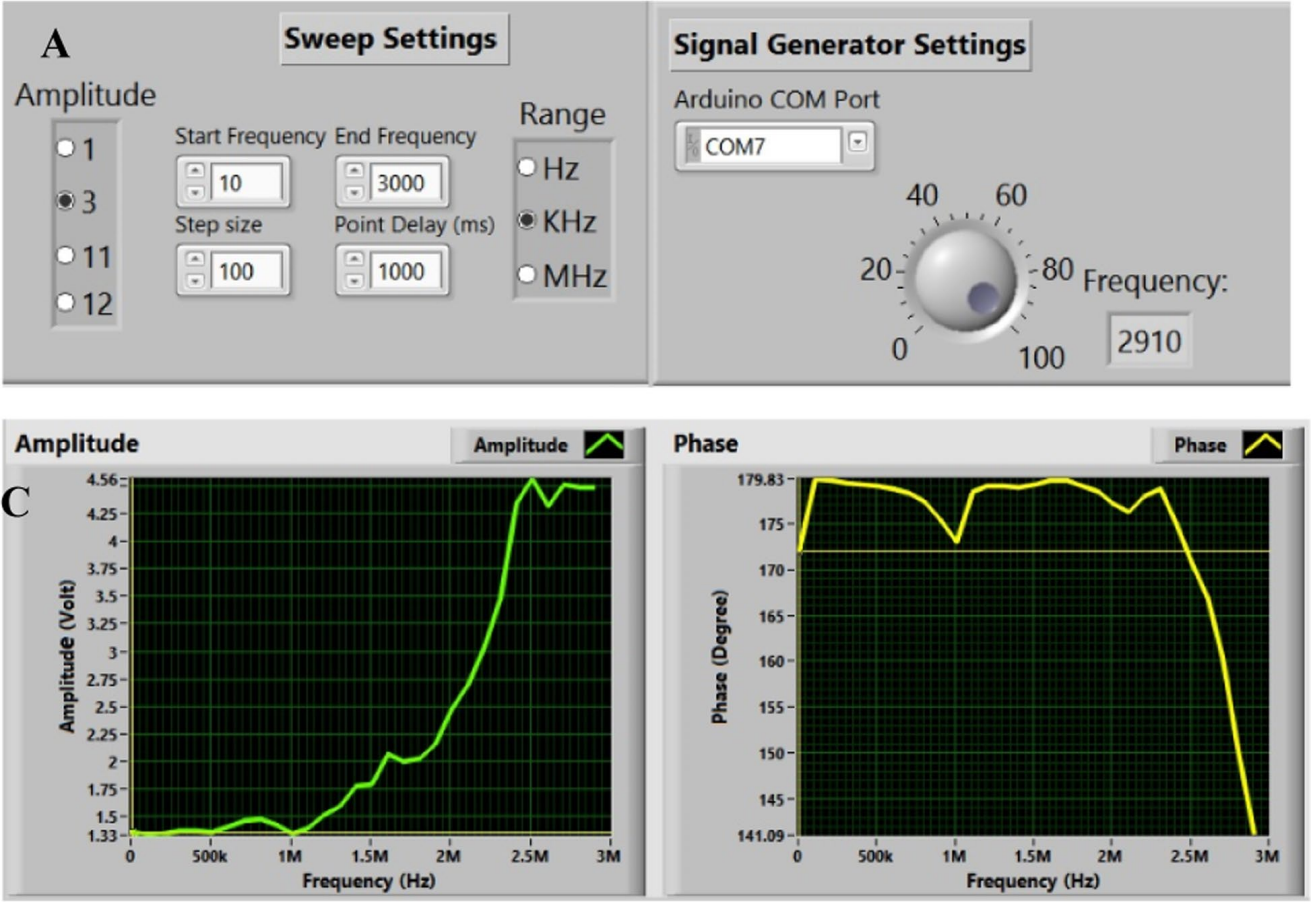
Illustrates the GUI that enables the user to interact with the microfluidic impedance system. As seen in **A** the GUI allows selecting and defining the connection port connecting to the Arduino microcontroller. **B** fig. shows the sweep settings panel, which sets the amplitude of the excitation signal and frequency range and specifies the sweeping frequency’s start and endpoints. Finally, **C** of the fig. displays the output signal features, including the amplitude and phase

This step attempts to provide a more user-friendly control system and software that overcomes the limitations of the conventional detecting system. This involves regulating the frequency, the excitation signal’s amplitude, the sensing signal’s amplitude, and the Experiment’s initiation. GUI, as shown in Fig. 5, is used to achieve the parameters controlling. The connection port that links to the Arduino microcontroller is defined and chosen in Fig. 5A. The sweep parameters panel, which is depicted in Fig. 5B, enables to specify of the excitation signal’s amplitude, frequency range, and start and end of the frequency spectrum. The phase and amplitude of the output signal are shown in Fig. 5C. For further analysis and signal processing using Matlab, the collected data will be stored as a CSV file. This file will include the input transmitted signal, desired output amplitude, phase difference, and sweeping frequency.

## Protocol for culturing and preparing non-viable and viable cells

The ATCC® HTB-22TM MCF7 cells were employed and grown in (DMEM), and then 10% bovine serum, 100 U/ml penicillin, and 100 mg/ml streptomycin were added. The cells were kept in a CO2 incubator with 5% CO2 at 37 degrees Celsius. The viable cell count was quantified using a hemocytometer and trypan blue staining while the cells were examined under an inverted microscope (Olympus IX70, USA).

## Electric field simulation

A numerical electric field simulation was carried out to comprehend the Experiment’s outcomes further. The interdigitated system was modeled using typical dimensions, refer to Method part, using COMSOL Multiphysics. Copper was chosen as the electrode material and was then coated with a silver layer (silver immersion for the sensor board), while FR-4 was selected as the substrate material. The strength of the (Eexc) was evaluated using the typical signal amplitude of 1.5 V that will be used in the experimental work to evaluate the output (Erec). The simulation results demonstrate the distribution and strength of the electric field that will be applied to the cells. The study’s strength lies in the correlation between the extracted experimental and simulation results.

**Fig. 6:**
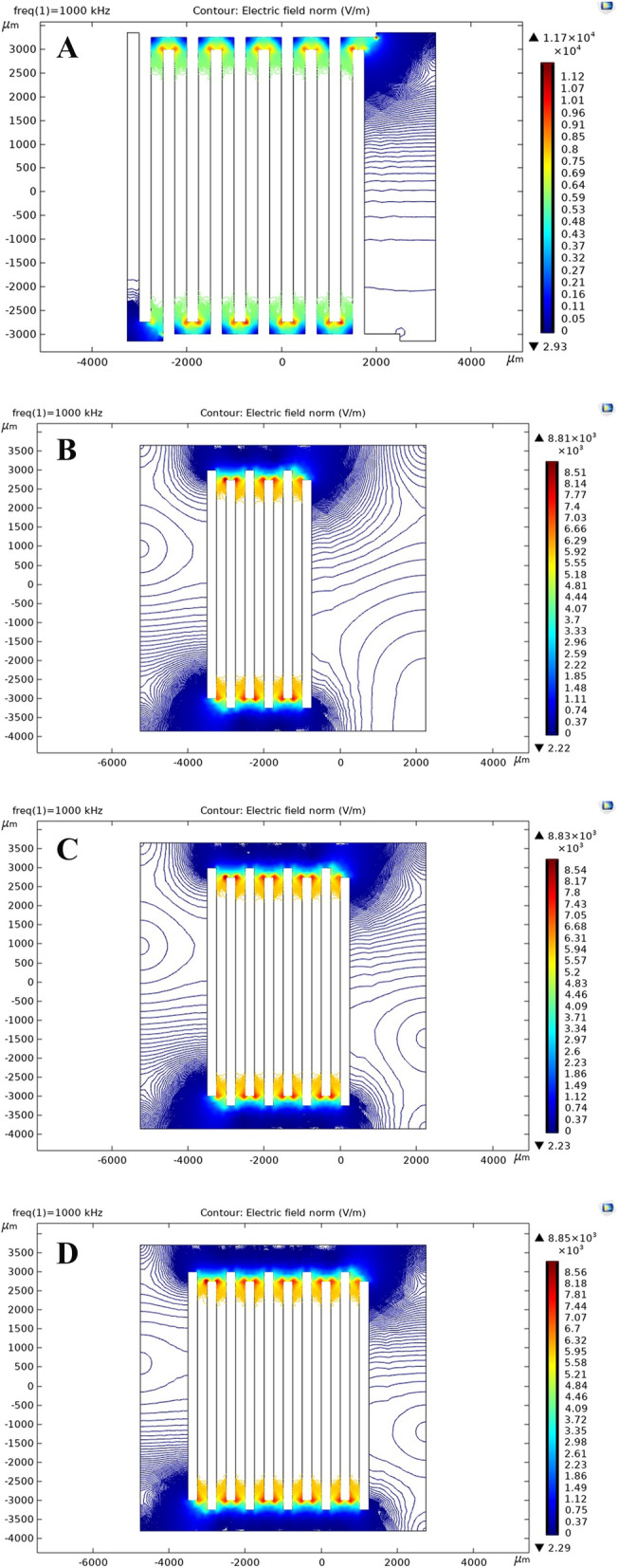
Depicts a map of the electric potential distribution as the number of electrodes increases with the electric field. **A** Shows the electric field distribution for the first configuration. **B** Shows the electric field map for the X1 configurations. **C** Shows the electric field for X2 configurations. Finally, **D** shows the response for the ten microelectrodes with the insulation layer

Figure 6 illustrates the electric field profiles for various combinations. The simulation results and contour figures indicate that the strength and direction of the non-uniform electric field are maximized at the edges of the microelectrodes. The extracted impedance from the experimental work can be used to define the influence of the electric field on the cells. The extracted electric fields from the different systems are presented in Table S2. Figure 6A shows that the maximum electric field is obtained with the interdigitated microelectrodes, and the interface track greatly influences the electric field compared to other configurations. The influence of the insulation layer can be seen in Fig. 6B, the first configuration with an insulation layer. Compared to Fig. 6A, the decrease in the electric field’s strength shows that the insulation layer may reduce the effect of leakage current. The influence of this modification can be tested by comparing the extracted impedance with different conditions and cells. The strength of the electric field increases at a nonlinear rate as the number of electrodes increases, as seen in Fig. 6C for six electrodes or in Fig. 6D for ten electrodes. The results demonstrate that the electric field with ten electrodes is lower than that extracted without an insulation layer by 23.5%.

## Extract the impedance using different configuration

### The experimental work requirements

The experimental work aims to understand the electrical behavior of cells when exposed to an electric field. One of the key characteristics is to study Beta dispersion, which is defined within the radio frequency range of 1 kHz to 10 MHz. This study focuses on the extraction of beta dispersion to characterize cell proliferation and viability. The total extracted impedance, Z vs. frequency (i.e., frequency spectrum), can be represented with a Bode plot. These plots demonstrate that the magnitude of Z decreases with an increase in frequency. The extracted impedance can be used to identify the combination of passive components R and C for the examined cell. The Bode plots were extracted for viable and non-viable MCF7 cells, with the concentrations recorded in Table S1. The study also includes the examination of the cell buffer solution, using DMEM for in vivo applications and PBS for in vitro applications, to evaluate the sensitivity of the whole system. Additionally, the study includes the analysis of two selected sizes of microbeads [10.4 µm and 24.9 µm], with the concentrations of the tested cells described in Table S1. The chosen volume for all cells and microbeads was 5 µl. The microbeads concentrations for the large size were ${10}^{5}cell$/5µL and ${10}^{6}cells$ /5µL for the small one. The difference between cells can be extracted at a specific value of frequency. The results demonstrate that the system enables high sensitivity to detect small cluster impedance, with the advantages of label-free measurements. The results define all possible techniques of electrode design to enhance the differentiation between cells based on size or cell viability.

## Results

### The extracted impedance

At this step, the system will be experimentally tested to confirm its functionality (i.e., test the response of the designed electrodes and plot the extracted impedance). The magnitude of the impedance can be calculated using Eq. 4:

4 $$\left|Z\right|=|{Z}_{cell}-{Z}_{without}|$$

Equation 4 describes the impedance (Z) difference between a cell and a sample without a cell. The magnitude of this difference (|Z|) represents the cell’s impedance. Avoiding the issues caused by fabrication asymmetry, all results are calculated using electrodes, not in contact with a cell as a reference.

**Fig. 7:**
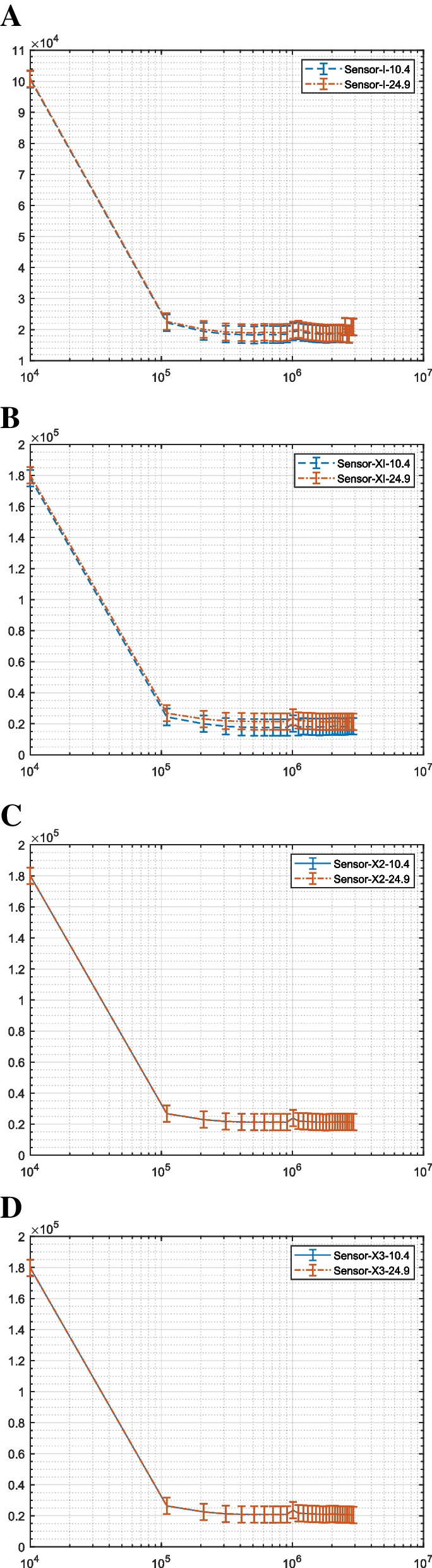
Illustrates the extracted impedance between two different sizes of microbeads as a function of frequency. **A** shows the response of the first configuration without an insulation layer. **B** illustrates the response of the first configuration of interdigitated microelectrodes (X1). **C** illustrates the response of the X2 configuration, and **D** illustrates the response of the X3 configuration. The insets display a specific frequency region from ${10}^{5}:{10}^{6}$ for each signal

Figure 7 illustrates the impedance response for different microbeads and their corresponding insets. The difference between the microbeads with the same dielectric properties is related to the polarization rate. The sensitivity of the system can be measured using microbeads as a reference. The numerical values for the real, imaginary, phase, and magnitude impedance at the different selected frequencies at a specific frequency value are presented and extracted in Table S3. The significant difference ΔΖ between the cells in the numerical form listed in Table S3 is used to define the ability of different configurations to enhance the differentiation between the cells’ polarization. The influence of the addition capacitance for the connection track (Fig. 7A) is primarily affected by the extracted impedance compared to the other configurations with the insulation layer (Fig. 7B).

5 $$\Delta Z=\frac{(|Zpbs-Zmedia|)}{0.5*(Zpbs+Zmedia)}*100\%$$

**Fig. 8:**
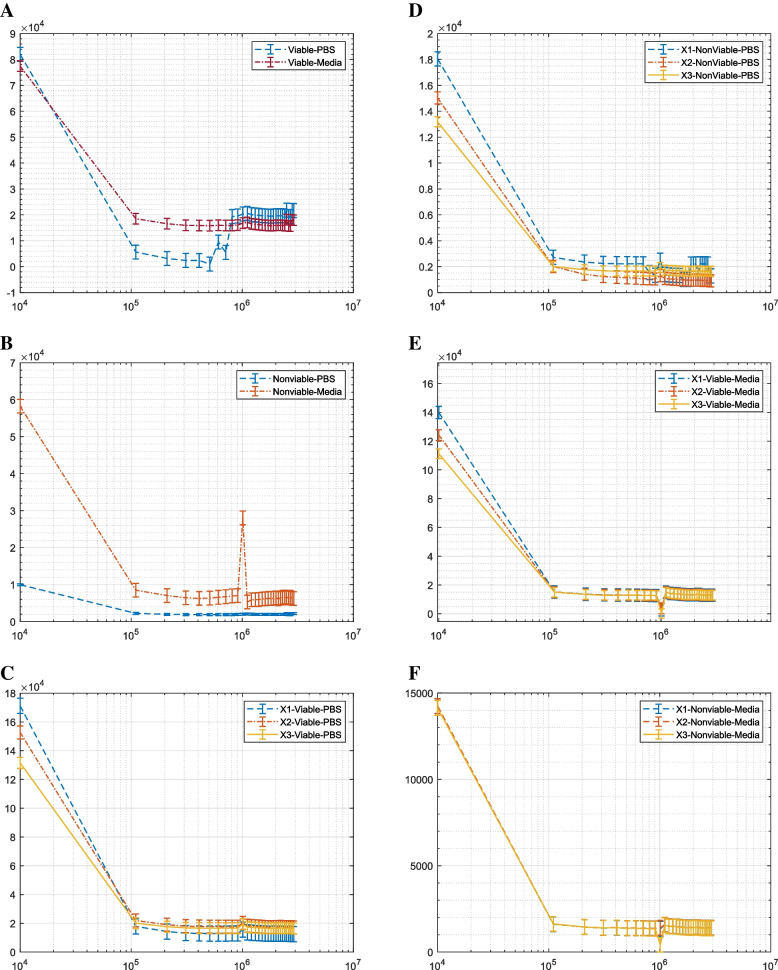
Illustrates the extracted impedance between various types of cells as a function of frequency. **A** The first configuration of interdigitated microelectrodes for viable cells with different buffer solutions is shown. **B** The measured impedance using the first configuration without an insulation layer with non-viable cells immersed in different buffer solutions is presented. **C** The response of different modified electrodes with different electrode numbers and an insulation layer when the cell is immersed in PBS is depicted. **D** The response of X1, X2, and X3 as modified electrodes with non-viable cells immersed in PBS solution is presented. **E** The modified electrode with viable cells immersed in media is shown. **F** The modified electrodes for non-viable cells immersed in media are illustrated. The insets display a specific selected region of frequency from ${10}^{5}:{10}^{6}$ at each signal

The increase in the difference, as depicted in Fig. 7B, illustrates how to avoid the influence of the leakage current that appeared in the output signal without the insulation layer in the first configuration. As shown in Fig. 7A, the decay in the difference between the cells can also be attributed to parasitic capacitance due to the increase in the parallel electrodes. The effect of the decay in the impedance based on the number of electrodes can be seen in Figs. 7 and 8. Table S3 provides the numerical difference between the microbeads at different frequencies, which can be expressed using Eq. 5 as $\Delta {Z}_{1-10Khz}$= 0.17%, $\Delta {Z}_{1-110Khz}$= 2.12%, ${\Delta Z}_{1-1.01Mhz}$= 5.30%, and $\Delta {Z}_{1-2.01Mhz}$= 6.01%. The differences between the microbeads were further intensified using the same number of electrodes with the insulation layer on the electrodes using X1 configurations, and the difference can be ${\Delta Z}_{2-10Khz}$= 0.60%, $\Delta {Z}_{2-110Khz}$= 22.85%, ${\Delta Z}_{2-1.01Mhz}$=5.25%, and $\Delta {Z}_{2-2.01Mhz}$= 11.71%. ∆Z1 has been measured using Sensor 1 without an insulation layer, and ∆Z2 using Sensor X1 with the same number of interdigitated electrodes but with an insulation layer. From this, it can be concluded that the insulation layer, combined with the optimized electrode number, can improve the differentiation between cells of different sizes. The next step is to examine these electrodes using in vivo and in vitro analysis with various buffer solutions and observe the cell viability behavior. Table S3 presents the numerical values for the impedance and phase of microbeads at specific frequency values using different configurations. These extracted values demonstrate the sensitivity of the overall system and the significant differences in various impedance components, including the real, imaginary, magnitude, and phase. These extracted features are important in analyzing cell differences based on cell viability, buffer solution type, cell size, and electrode configurations.

### Extracting the impedance of breast cancer cells using the first configuration

Different configurations are used to extract the impedance between cells based on cell viability and different buffer solutions. As the conductivity of the media is greater than the conductivity of PBS, the impedance of cells in DMEM media is expected to be lower than that of the cells immersed in PBS. The impedance spectrum supports this concept in Fig. 8A, which uses the first configuration without an insulation layer. Furthermore, numerical extraction at different frequencies listed in Table S4 is also used, utilizing the percentage difference as seen in Eq. 5. It was found that for the interdigitated electrodes without the insulation layer, $\Delta {Z}_{1V-10Khz}$= 16.90% and $\Delta {Z}_{1V-1.01Mhz}$= 6.19%. Here, $\Delta {Z}_{1V}$ refers to the difference between viable cells with different buffer solutions using the first configuration (i.e., sensor one). This system can differentiate between viable cells with different buffer solutions. Another factor is that the components, such as vitamins of the DMEM solution, are greater than those of the PBS solution, as the latter is considered component-free compared to DMEM; thus, the number of components increases as the extracted impedance decreases. As the DMEM enhances the cell’s continuous feeding compared to PBS solution, this influence can be observed by the compression between the non-viable cells in Fig. 8A. Unstable cell behavior can be observed in Fig. 8A due to leakage current. The results for the impedance using the interdigitated electrodes with different buffer solutions are described in Fig. 8B. These results match the previous analysis for the capability of interdigitated electrodes to extract the difference between viable and non-viable cells compared to conventional planar electrodes [25]. The decay in the impedance between the viable and non-viable cells is evident in Fig. 8A and B. $\Delta {Z}_{1NV-10Khz}$= 151.44%, $\Delta {Z}_{1NV-1.01Mhz}$= 177.42%. Here, $\Delta {Z}_{1NV}$ refers to the difference between the non-viable cells with different buffer solutions using the first configuration sensor 1. The media preserves the viability of the cells in comparison to PBS. The ability of sensor 1 to differentiate between the viable and non-viable cells when immersed in PBS or DMEM media will be described with the repeatability test in the statistical analysis paragraph.

### Extracting the impedance of breast Cancer cells using the electrodes with the insulation layer

This section tested the effect of using an insulation layer on different configurations using MCF7 cells and various buffer solutions. The results of the viable cells tested using the selected configuration are presented in Fig. 8C, D, E, and F, where the cells were immersed in PBS. The Bode plot in Fig. 8C represents the viable cells, and Fig. 8D represents the non-viable cells. The numerical values can be found in table S4. Figure 8E and F showed the impedance spectrum when the cells were immersed in PBS and DMEM, respectively. Figure 8C and E indicate that the fluctuation in the impedance spectrum as a function of the number of sensing electrodes directly influences the viable cells. Non-viable cells immersed in PBS (Fig. 8D) and non-viable cells immersed in DMEM (Fig. 8F) show an enhanced response. The numerical values for the extracted impedance, using the percentage difference as Eq. 5, showed that ∆Z_(x1V-10 kHz) = 1.51%, ∆Z_(x1V-1.01 MHz) = 162.02%, where ∆Z_x1V refers to the difference between the viable cells with different buffer solutions using the first modified configuration with the insulation layer sensor X1. Similarly, ∆Z_(x1NV-10 kHz)% = 32.27%, ∆Z_(x1NV-1.01 MHz)% = 57.28%, refers to the difference between the non-viable cells with different buffer solutions using the same sensor X1. Furthermore, ∆Z_(× 2-V-10 kHz)% = 173.03%, ∆Z_(× 2-V-1.01 MHz) = 11.39%, where ∆Z_(× 2-V) refers to the difference between the viable cells with different buffer solutions using the second modified configuration with an increased number of electrodes (sensor X2). The results indicate that the system’s stability in extracting the significant difference between the cells with different numbers of electrodes increases at low frequencies. Similarly, ∆Z_(× 2-NV-10 kHz) = 86.14%, ∆Z_(× 2-NV-1.01 MHz) = 36.17%, ∆Z_(× 2-NV) refers to the difference between the non-viable cells with different buffer solutions using X2. Additionally, the analysis data for X3 can be described as ∆Z_(× 3-V-10 kHz)% = 102.40%, ∆Z_(× 3-V-1.01 MHz) = 85.81%. ∆Z_(× 3-NV-10 kHz) = 35.26%, ∆Z_(× 3-NV-1.01 MHz) = 12.53%. The ability of sensor 1 to extract the response of different buffer solutions with non-viable cells was clearly shown as a percentage value, and the enhancement using the insulation layer for viable cells can be seen when comparing sensor 1 with different electrodes with an insulation layer and different electrode numbers X1, X2, and X3. The results also showed that when the non-viable cells were immersed in PBS solution (Fig. 8B and D), the sensing signal mainly depended on the cell’s polarization. However, when the non-viable cells were immersed in DMEM, the output signal was mainly affected by the surrounding buffer solution components (Fig. 8B and F). The ability of the modified electrodes with the insulation layer to differentiate between viable and non-viable cells when immersed in PBS or DMEM media will be described with the repeatability test in the statistical analysis paragraph, especially the comparison between sensor one and sensor X1. Table S4 presents the numerical values for the impedance and phase of MCF7 cells at specific frequency values using different configurations. The focus of this Table S4 is on the effect of buffer solution and cell viability on the impedance of breast cancer cells.

## Statistical analysis of electrical impedance

### The peak Value

The peak values (real and imaginary impedance component, magnitude, and phase angle) for injecting microbeads are shown in Fig. S1 as the results of impedance analysis. The extracted values are the average of ten samples of repeatability tests for the microbeads and ten samples for the MCF7 cells. The extracted values of targeted cells, including the error bars (maximum and minimum), were successfully separated at specific frequencies for each signal of the targeted cells. Figure S1A displays the difference between the two microbead sizes, with the enhancement in contrast evident in Fig. S1B, as a function of the modified insulation layer, which reduces the influence of leakage current. The same sensors were used to extract the difference in impedance between different clusters of MCF7 viable and non-viable cells when immersed in PBS and media buffers. Figure S2 shows the peak values of electrical impedance responses of MCF7 cells as a function of frequency. The responses are measured for the magnitude part of the impedance and are shown for four different conditions. In Fig. S2, four different conditions are shown as the result of impedance analysis. Figure S2A displays the results of using Sensor 1 with PBS as the buffer solution. Figure S2B shows the results of using Sensor X1 with PBS as the buffer solution. Figure S2C illustrates the results of using Sensor 1 with DMEM as the buffer solution, and Fig. 2D shows the results of using Sensor X1 with DMEM as the buffer solution. The vertical bars in the graph represent the error, which is defined by the maximum and minimum values.

### The Differentiation Index (DI)

The Differentiation Index (DI) is established by measuring the electrical impedances at various frequencies to evaluate the effectiveness of differentiation between the target cells objectively. The formula for DI is:

6 $$DI=\frac{Gap}{{D}_{AVG}}=\frac{{U}_{MIN}-{L}_{MAX}}{{U}_{AVG}-{L}_{AVG}}$$

where $Gap$,${D}_{AVG}$,${U}_{AVG}$, ${U}_{MIN}$, ${L}_{AVG}$, and ${L}_{MAX}$ represent the gap, average difference, and average value of the upper level, the minimum value of the upper level, the average value of the lower level, and the maximum value of the lower level, respectively.

**Fig. 9:**
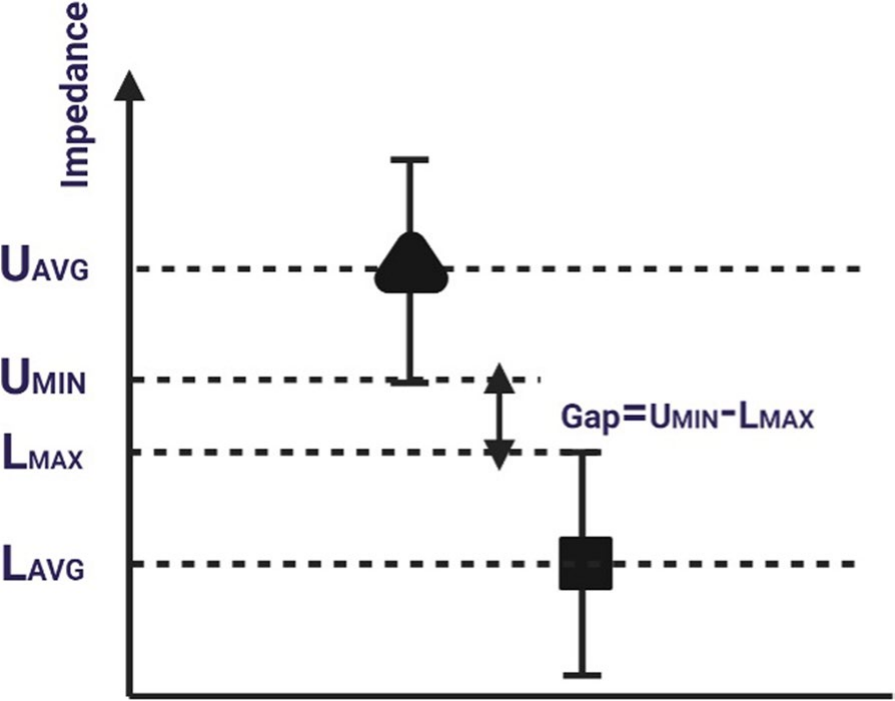
Definition of the differentiation index as a nondimensional feature

As illustrated in Fig. 9, the broadest gap and narrowest average difference are necessary to ensure the highest value of DI, indicating that the targeted cells are well-distinguished with low variance. The differentiation indexes for the measured electrical impedances are summarized in Tables S5, S6, and S7. A negative differentiation index represents specific frequencies at which the electrical impedances of the targeted cells overlap, making it difficult to differentiate them. The signs of the differentiation indexes are positive for several extracted values using the proposed system. Table S5 defines the differentiation index for the microbeads using Sensor 1 and Sensor X1, allowing for the clear distinction between cells with the positive index at various frequencies. Table S5 describes the differentiation index for both selected sensors when the target cells are MCF7 immersed in PBS, while Table S7 describes the same when immersed in MDEM media.

The results presented in Tables S5, S6, and S7 indicate that the system can accurately distinguish between cells based on their electrical impedance properties, as demonstrated by the Differentiation Index (DI) values at each frequency. The DI values reveal that the system effectively differentiates between cells regarding the impedance’s real, imaginary, magnitude, and phase components. Additionally, the results demonstrate that the system can detect differences between cells at high frequencies, such as 2.01 MHz. The results confirm the system’s effectiveness in differentiating between cells based on their electrical impedance properties.

### The reliability of the proposed overall system

The reliability of the proposed overall system design for impedance spectroscopy can be calculated using Eq. 7, which states that reliability is given by.

7 $$1-\left|\frac{{Z}_{max}-{Z}_{min}}{{Z}_{avg}}\right|*100\mathrm{\%}$$

where ${Z}_{max}, {Z}_{min},and {Z}_{avg}$ are the maximum, minimum, and average impedance values, respectively. Data analysis for two selected passive components, ${R}_{1}$=4KΩ and ${R}_{2}$=20KΩ, found that the reliability equals 98.03% and 98.49%, respectively.

## Conclusions

In this research, a portable and easy-to-use system is designed, fabricated, and experimentally tested. This system includes various sensor configurations. The system determines the dielectric parameters of Microbeads and MCF7 cells in different buffer solutions. The readout circuit is demonstrated, discussed, and shows a high sensitivity, which allows the extraction of a significant difference between viable and dead cells. The system could extract and differentiate between cells based on their size using two polystyrene microbead cell types. AMicrofluidic chamber is used to avoid contamination. The study was applied to cells in different buffer solutions, such as PBS and DMEM, to enhance in-vivo and in-vitro analysis. The paper discussed and demonstrated the effect of modifying the sensing electrodes with an insulation layer, which can prevent leakage current. The high sensitivity and stability of the measurement system exhibit minimal fluctuation in the impedance of the cluster after insulation modifications. The results show that viable cells’ impedance is greater than non-viable cells. Phosphate buffer solution enhances the difference between viable and non-viable cells in the first system configuration without an insulation layer. This difference decreases when the cell is immersed in DMEM. However, this influence can be avoided by using the insulation layer as described in other selected configurations. Compared to previous studies, the proposed system focuses on extracting the cell’s impedance without utilizing dielectrophoresis force, which overcomes the influence of cell position on the extracted impedance.

The results of the experiments are in good agreement with the simulation results. Additionally, the interdigitated electrodes provide coplanar excitation in the 2D plane, and the design avoids the complexity of 3D designs. The study also shows the optimal number of interdigitated electrodes that can be used to extract variations in dielectric parameters of cells without leakage current or parasitic capacitance. The advantage of interdigitated electrodes is that they allow the cell to be inserted between the source and sink electrodes, thus confining most of the electric field to the cell membrane, which contains meaningful electrical properties. Additionally, the variance of the measured values can be reduced because the targeted cells are in almost the same position between the source and sink electrodes in every cell assay. The system effectively defined breast cancer cells in different buffer solutions and showed appropriate conditions for the number of electrodes under test conditions. The results show that the insulation layer enhances the difference between cells based on size. The experimental results match the Mossotti factor response without any shift in the characteristic frequency. The measured differentiation indexes demonstrate the system’s ability to define the difference between cells at different frequencies.

## Supplementary Information

**Additional file 1: Table S1.** Concentration of the cells used in the experiments. **Table S2.** Simulation results for the electric field using the different systems. **Table S3.** The extracted numerical values for the impedance of microbeads and phase at specific values of frequency using the different configurations. **Table S4.** The extracted numerical values for the impedance and phase of MCF7 at specific values of frequency using the different configurations. **Table S5.** Differentiation indexes for electrical impedances for microbeads concerning measured frequency. **Table S6.** Differentiation indexes for electrical impedances for MCF7 in PBS concerning measured frequency. **Table S7.** Differentiation indexes for electrical impedances for MCF7 in DMEM concerning measured frequency. **Fig. S1.** Peak Value of Electrical impedance responses of Microbeads as a function of frequency for magnitude part at the different frequency Fig. S1A) Sensor 1. Fig. S1B) Modified electrodes with the same number X1. The vertical bars represent the error defined by maximum and minimum values. **Fig. S2.** Peak Value of Electrical impedance responses of MCF7 as a function of frequency for magnitude part at the different frequency Fig. S2A) Sensor 1 vs. PBS as a buffer solution. Fig. S2B) Sensor X1 vs. PBS as a buffer solution. Fig. S2C) using Sensor 1 when DMEM is the buffer solution. Fig. S2D) Sensor x1 for DMEM as the buffer solution. The vertical bars represent the error defined by maximum and minimum values.

## Data Availability

All relevant data are contained within the article.

## Abbreviations

Z: The impedance
DI: Differentiation Index
DMEM: Dulbecco’s modified Eagle’s medium
PBS: Phosphate buffer saline
MCF7: Early-stage human breast cancer cell
GUI: Graphical User Interface
AC: Alternating current
FACS: Fluorescence Activated Cell Sorting
MACS: Magnetic Activated Cell Sorting
DDS: Digital Synthesizer AD9851

## References

[1] Azamjah N, Soltan-Zadeh Y, Zayeri F (2019). Global trend of breast cancer mortality rate: a 25-year study. Asian Pac J Cancer Prev.

[2] Pradhan R, Rajput S, Mandal M, Mitra A, Das S (2014). Frequency dependent impedimetric cytotoxic evaluation of anticancer drug on breast cancer cell. Biosens Bioelectron.

[3] Makki J (2015). Diversity of breast carcinoma: histological subtypes and clinical relevance. Clin Med Insights Pathol.

[4] Ducatman BS, Wang HH (2014). Breast. Cytology: diagnostic principles and clinical correlates.

[5] Catanzariti F, Avendano D, Cicero G, Garza-Montemayor M, Sofia C, VenanziRullo E (2021). High-risk lesions of the breast: concurrent diagnostic tools and management recommendations. Insights into Imaging..

[6] Sgroi DC (2010). Preinvasive breast cancer. Annu Rev Pathol.

[7] Łukasiewicz S, Czeczelewski M, Forma A, Baj J, Sitarz R, Stanisławek A (2021). Breast Cancer—epidemiology, risk factors, classification, prognostic markers, and current treatment strategies—an updated review. Cancers..

[8] Lovitt CJ, Shelper TB, Avery VM (2014). Advanced cell culture techniques for cancer drug discovery. Biology (Basel).

[9] Balik K, Matulewicz K, Modrakowska P, Kowalska J, Montane X, Tylkowski B, Bajek A. 4 Advanced cell culture techniques for cancer research. Medical Physics: Models and Technologies in Cancer Research, edited by Anna Bajek and Bartosz Tylkowski, Berlin, Boston: De Gruyter; 2021, p. 81-102. 10.1515/9783110662306-004.

[10] He Z, Chen Z, Tan M, Elingarami S, Liu Y, Li T (2020). A review on methods for diagnosis of breast cancer cells and tissues. Cell Prolif.

[11] Pandey S, Mehendale N, Paul D. Single-Cell Separation. In: Handbook of Single Cell Technologies; 2018. p. 1–28.

[12] Zhang X, Powell K, Li L (2020). Breast Cancer stem cells: biomarkers, identification and isolation methods, regulating mechanisms, cellular origin, and beyond. Cancers (Basel).

[13] Bargahi N, Ghasemali S, Jahandar-Lashaki S, Nazari A (2022). Recent advances for cancer detection and treatment by microfluidic technology, review and update. Biol Proced Online.

[14] Gökçe F, Ravaynia PS, Modena MM, Hierlemann A (2021). What is the future of electrical impedance spectroscopy in flow cytometry?. Biomicrofluidics.

[15] Zhuang J, Schoenbach KH, Kolb JF. Time domain dielectric spectroscopy of biological cells after pulsed electric field exposure. In: Annual Report - Conference on Electrical Insulation and Dielectric Phenomena, CEIDP; 2011. p. 44–7.

[16] Faley S, Seale K, Hughey J, Schaffer DK, Vancompernolle S, McKinney B (2008). Microfluidic platform for real-time signaling analysis of multiple single T cells in parallel. Lab Chip.

[17] Zhang Z, Zheng T, Zhu R (2020). Single-cell individualized electroporation with real-time impedance monitoring using a microelectrode array chip. Microsyst Nanoeng..

[18] Le Ngoc HT, Kim J, Park J, Cho S (2019). A review of electrical impedance characterization of cells for label-free and real-time assays. Biochip J.

[19] Desai SP, Coston A, Berlin A (2019). Micro-electrical impedance spectroscopy and identification of patient-derived dissociated tumor cells. IEEE Trans Nanobioscience.

[20] Huang X, Nguyen D, Greve DW, Domach MM (2004). Simulation of microelectrode impedance changes due to cell growth. IEEE Sens J.

[21] Jang LS, Wang MH (2007). Microfluidic device for cell capture and impedance measurement. Biomed Microdevices.

[22] Buscaglia LA, Oliveira ON, Carmo JP (2021). Roadmap for electrical impedance spectroscopy for sensing: a tutorial. IEEE Sens J.

[23] Miklavčič D, Pavšelj N, Hart FX (2006). Electric Properties of Tissues. Wiley Encycl Biomed Eng.

[24] Mansoorifar A, Koklu A, Ma S, Raj GV, Beskok A (2018). Electrical impedance measurements of biological cells in response to external stimuli. Anal Chem..

[25] Sherif S, Morsy OE, Ziko L, Siam R, Ghallab YH, Ismail Y (2020). Integration of tri-polar microelectrodes for performance enhancement of an impedance biosensor. Sens Biosensing Res.

[26] Gerasimenko T, Nikulin S, Zakharova G, Poloznikov A, Petrov V, Baranova A (2019). Impedance spectroscopy as a tool for monitoring performance in 3D models of epithelial tissues. Front Bioeng Biotechnol.

[27] Eker B, Meissner R, Bertsch A, Mehta K, Renaud P (2013). Label-free recognition of drug resistance via impedimetric screening of breast Cancer cells. PLoS One.

[28] Sun T, Morgan H. Single-cell microfluidic impedance cytometry: a review. Microfluid Nanofluid. 2010;8:423–43. 10.1007/s10404-010-0580-9.

[29] Chen L, Han Z, Fan X, Zhang S, Wang J, Duan X (2020). An impedance-coupled microfluidic device for single-cell analysis of primary cell wall regeneration. Biosens Bioelectron.

[30] Li P, Highfield PE, Lang ZQ, Kell D (2021). Cervical Cancer prognosis and diagnosis using electrical impedance spectroscopy. J Electr Bioimpedance.

[31] Shaw AD, Winson MK, Woodward AM, McGovern AC, Davey HM, Kaderbhai N (2000). Rapid analysis of high-dimensional bioprocesses using multivariate spectroscopies and advanced chemometrics. Adv Biochem Eng Biotechnol.

[32] Pan Y, Jiang D, Gu C, Qiu Y, Wan H, Wang P (2020). 3D microgroove electrical impedance sensing to examine 3D cell cultures for antineoplastic drug assessment. Microsyst Nanoeng.

[33] Shah P, Zhu X, Zhang X, He J, Li CZ (2016). Microelectromechanical system-based sensing arrays for comparative in vitro nanotoxicity assessment at single cell and small cell-population using electrochemical impedance spectroscopy. ACS Appl Mater Interfaces.

[34] Du X, Kong J, Liu Y, Xu Q, Wang K, Huang D (2021). The measurement and analysis of impedance response of hela cells to distinct chemotherapy drugs. Micromachines (Basel).

[35] Abdolahad M, Janmaleki M, Taghinejad M, Taghnejad H, Salehi F, Mohajerzadeh S (2013). Single-cell resolution diagnosis of cancer cells by carbon nanotube electrical spectroscopy. Nanoscale.

[36] Pires NMM, Dong T, Yang Z, Høivik N, Zhao X (2011). A mediator embedded micro-immunosensing unit for electrochemical detection on viruses within physiological saline media. J Micromech Microeng.

[37] Turcan I, Olariu MA (2020). Dielectrophoretic manipulation of Cancer cells and their electrical characterization. ACS Comb Sci.

[38] Zimmermann J, van Rienen U (2021). Ambiguity in the interpretation of the low-frequency dielectric properties of biological tissues. Bioelectrochemistry.

[39] Stoneman MR, Kosempa M, Gregory WD, Gregory CW, Marx JJ, Mikkelson W (2007). Correction of electrode polarization contributions to the dielectric properties of normal and cancerous breast tissues at audio/radiofrequencies. Phys Med Biol.

[40] Amini M, Hisdal J, Kalvøy H (2018). Applications of bioimpedance measurement techniques in tissue engineering. J Electr Bioimpedance.

[41] Sherif S, Morsy OE, Ziko L, Siam R, Ghallab YH, Ismail Y. Microfluidic platform for monitoring the dielectric parameters of U2OS cells. In: Proceedings of the International Conference on Microelectronics, ICM; 2019. p. 53–6.

[42] Wang MH, Chang WH (2015). Effect of electrode shape on impedance of single HeLa cell: a COMSOL simulation. Biomed Res Int..

[43] Abdur Rahman AR, Price DT, Bhansali S (2007). Effect of electrode geometry on the impedance evaluation of tissue and cell culture. Sens Actuators B Chem.

[44] Wang HC, Nguyen NV, Lin RY, Jen CP (2017). Characterizing esophageal cancerous cells at different stages using the dielectrophoretic impedance measurement method in a microchip. Sensors.

[45] Aslam MA, Riaz K, Saleem MM (2021). Gradient-based impedance synthesis for breast and lung cancer cell screening deploying planar and nano-structured electrodes. Med Biol Eng Comput.

[46] Piyasena ME, Graves SW (2014). The intersection of flow cytometry with microfluidics and microfabrication. Lab Chip.

[47] Kim HW, Park Y, Yun J, Lim J, Lee JZ, Shin DG, et al. Differentiation Between Normal and Cancerous Human Urothelial Cell Lines Using Micro-Electrical Impedance Spectroscopy at Multiple Frequencies. J Med Biol Eng. 2019;39:86–95. 10.1007/s40846-018-0426-6.

[48] Park I, Nguyen T, Park J, Yoo AY, Park JK, Cho S (2018). Impedance characterization of chitosan cytotoxicity to MCF-7 breast Cancer cells using a multidisc indium tin oxide microelectrode array. J Electrochem Soc.

[49] Caselli F, de Ninno A, Reale R, Businaro L, Bisegna P (2018). A novel wiring scheme for standard chips enabling high-accuracy impedance cytometry. Sens Actuators B Chem.

[50] Sherif S, Ghallab YH, El-Wakad MT, Ismail Y. Impedance spectroscopy based on the cell trajectory and new strategy to enhance the accuracy of the detection in the microfluidic system. In: Proceedings of the International Conference on Microelectronics, ICM; 2021. p. 106–9.

[51] Gómez-Sánchez JA, Bertemes Filho P. Saline solution impedance sensor based on poly methyl methacrylate and graphite: preliminary results. J. Phys.: Conf. Ser. Volume1272 012025.

[52] Grossi M, Riccò B (2017). Electrical impedance spectroscopy (EIS) for biological analysis and food characterization: a review. J Sens Sens Syst.

[53] Beltrán-Pitarch B, Prado-Gonjal J, Powell AV, Martínez-Julián F, García-Cañadas J (2019). Complete characterization of thermoelectric materials by impedance spectroscopy. J Phys Chem C.

[54] Liu Q. The influence of temperature on the impedance characteristics of PEMFC. J Phys Conf Ser. 2020;1605.

[55] HuberOskooeiCasadevallSolvasDemelloKaigala DAXAGV (2018). Hydrodynamics in cell studies. Chem Rev.

[56] Sherif S, Ghallab YH, El-Wakad MT, Ismail Y (2022). A Novel stimulation and impedance sensing setup for dielectrophoresis based microfluidic platform. Alex Eng J.

[57] Jang LS, Huang PH, Lan KC (2009). Single-cell trapping utilizing negative dielectrophoretic quadrupole and microwell electrodes. Biosens Bioelectron.

[58] Swami P, Sharma A, Anand S, Gupta S (2021). DEPIS: A combined dielectrophoresis and impedance spectroscopy platform for rapid cell viability and antimicrobial susceptibility analysis. Biosens Bioelectron.

[59] Schalenbach M, Durmus YE, Tempel H, Kungl H, Eichel RA (2021). Double layer capacitances analysed with impedance spectroscopy and cyclic voltammetry: validity and limits of the constant phase element parameterization. Phys Chem Chem Phys.

[60] Amor YB, Sutter EMM, Takenouti H, Orazem ME, Tribollet B (2014). Interpretation of electrochemical impedance for corrosion of a coated silver film in terms of a pore-in-pore model. J Electrochem Soc.

[61] Lakatos-Varsányi M, Furko M, Pozman T (2011). Electrochemical impedance spectroscopy study on silver coated metallic implants. Electrochim Acta.

[62] Turcan I, Caras I, Schreiner TG, Tucureanu C, Salageanu A, Vasile V (2021). Dielectrophoretic and electrical impedance differentiation of cancerous cells based on biophysical phenotype. Biosensors (Basel).

[63] Wolf M, Gulich R, Lunkenheimer P, Loidl A (2011). Broadband dielectric spectroscopy on human blood. Biochim Biophys Acta Gen Subj.

