# Supplementary material for: Optimization design of interdigitated microelectrodes with an insulation layer on the connection tracks to enhance efficiency of assessment of the cell viability
Source: BMC Biomed Eng. 2023 May 1;5:4. doi: 10.1186/s42490-023-00070-w (PMC10150490; doi:10.1186/s42490-023-00070-w)
Supplement: Supplementary file 1 — Additional file 1: Table S1. Concentration of the cells used in the experiments. Table S2. Simulation results for the electric field using the different systems. Table S3. The extracted numerical values for the impedance of microbeads and phase at specific values of frequency using the different configurations. Table S4. The extracted numerical values for the impedance and phase of MCF7 at specific values of frequency using the different configurations. Table S5. Differentiation indexes for electrical impedances for microbeads concerning measured frequency. Table S6. Differentiation indexes for electrical impedances for MCF7 in PBS concerning measured frequency. Table S7. Differentiation indexes for electrical impedances for MCF7 in DMEM concerning measured frequency. Fig. S1. Peak Value of Electrical impedance responses of Microbeads as a function of frequency for magnitude part at the different frequency Fig. S1A) Sensor 1. Fig. S1B) Modified electrodes with the same number X1. The vertical bars represent the error defined by maximum and minimum values. Fig. S2. Peak Value of Electrical impedance responses of MCF7 as a function of frequency for magnitude part at the different frequency Fig. S2A) Sensor 1 vs. PBS as a buffer solution. Fig. S2B) Sensor X1 vs. PBS as a buffer solution. Fig. S2C) using Sensor 1 when DMEM is the buffer solution. Fig. S2D) Sensor x1 for DMEM as the buffer solution. The vertical bars represent the error defined by maximum and minimum values. [file 42490_2023_70_MOESM1_ESM.docx]

Supplementary Information. The Experiment Results and The Statistical Analysis

Table S1 presents the concentration of cells and buffer solutions used in experiments. Table S2 shows simulation results for the electric field using different systems. Table S3 displays numerical values for the impedance and phase of microbeads at specific frequency values using different configurations. The results help analyze cell differences based on cell viability, buffer solution type, cell size, and electrode configurations. Table S4 presents numerical values for the impedance and phase of MCF7 cells at specific frequency values using different structures, focusing on the effect of buffer solution and viability on breast cancer cells. The Differentiation Index (DI) [Tables S5, S6, and S7] is a measure of the electrical impedance at a particular frequency used to evaluate the ability to differentiate between target cells objectively; it can be used to assess the effectiveness of differentiation between cells and can provide valuable insights for various applications such as new cell therapies or monitoring existing treatments.

Table S1 Concentration of the cells used in the experiments.

| **Type of cells** | **Cell count & comments** | **Suspended in solution** |
| --- | --- | --- |
| MCF7 (alive) | 3,200,000cells/ml | DMEM Media |
| MCF7 (Dead) | 1,960,000cells/ml | DMEM Media |
| MCF7 (alive) | 2,730,000cells/ml | PBS |
| MCF7 (Dead) | 1,300,000cells/ml | PBS |

Table S2 Simulation results for the electric field using the different systems.

| **System** | **MAX. Electric Field NormV/m** |
| --- | --- |
| System I | ${1.12*10}^{4}$ |
| System X1 | ${8.51*10}^{3}$ |
| System X2 | ${8.54*10}^{3}$ |
| System X3 | ${8.56*10}^{3}$ |

Table S3: The extracted numerical values for the impedance of microbeads and phase at specific values of frequency using the different configurations

| **Beads10.4µm** | **Sensor1** | | | |
| --- | --- | --- | --- | --- |
| **Frequency** | **Real** | **Imag.** | **Mag.** | **Phase.** |
| 10Khz | 5.5418e+04 | -8.4102e+04 | 1.0063e+05 | -2.4783e+01 |
| 110Khz | 2.3042e+04 | -3.8942e+02 | 2.2223e+04 | -7.6197e+01 |
| 1.01Mhz | 1.5259e+04 | -1.1854e+04 | 1.9320e+04 | -2.9453 |
| 1.51Mhz | 1.2242e+02 | -1.8710e+04 | 1.8692e+04 | -8.0553 |
| 2.01Mhz | 1.2590e+04 | -1.3831e+04 | 1.8670e+04 | -1.0839e+01 |

| **Beads24.9µm** | **Sensor1** | | | |
| --- | --- | --- | --- | --- |
| **Frequency** | **Real** | **Imag.** | **Mag.** | **Phase.** |
| 10Khz | 5.5322e+04 | -8.4366e+04 | 1.0087e+05 | -1.3762e+01 |
| 110Khz | 2.2558e+04 | -1.3409e+03 | 2.2597e+04 | -3.5440 |
| 1.01Mhz | 1.6089e+04 | -1.1816e+04 | 1.9864e+04 | -2.3192e+01 |
| 1.51Mhz | 1.1492e+03 | -2.0366e+04 | 1.9118e+04 | -8.8386e+01 |
| 2.01Mhz | 1.3370e+04 | -1.3546e+04 | 1.8952e+04 | -1.8997e+01 |

| **Beads10.4µm** | **SensorX1** | | | |
| --- | --- | --- | --- | --- |
| **Frequency** | **Real** | **Imag.** | **Mag.** | **Phase.** |
| 10Khz | 5.9287e+04 | -1.6814e+05 | 1.7828e+05 | -5.0291 |
| 110Khz | 9.8522e+03 | -2.2807e+04 | 2.4372e+04 | -2.9881e+01 |
| 1.01Mhz | 2.3999e+04 | -3.7315e+03 | 2.0193e+04 | -7.2503e+01 |
| 1.51Mhz | 1.7972e+04 | -2.2477e+03 | 1.7930e+04 | -1.3993e+01 |
| 2.01Mhz | 1.7457e+04 | -6.9627e+03 | 1.8272e+04 | -2.5556e+01 |

| **Beads24.9µm** | **SensorX1** | | | |
| --- | --- | --- | --- | --- |
| **Frequency** | **Real** | **Imag.** | **Mag.** | **Phase.** |
| 10Khz | 5.9646e+04 | -1.7026e+05 | 1.8010e+05 | -5.3924e+01 |
| 110Khz | 1.2394e+04 | -2.3811e+04 | 2.6806e+04 | - 1.8146e+01 |
| 1.01Mhz | 2.5292e+04 | - 1.1687 | 2.4094e+04 | - 8.6689e+01 |
| 1.51Mhz | 2.1925e+04 | -3.0060e+03 | 2.1421e+04 | - 6.0320e+01 |
| 2.01Mhz | 1.9628e+04 | -9.1801e+03 | 2.1288e+04 | - 4.2870e+01 |

| **Beads10.4µm** | **SensorX2** | | | |
| --- | --- | --- | --- | --- |
| **Frequency** | **Real** | **Imag.** | **Mag.** | **Phase.** |
| 10Khz | 5.9778e+04 | -1.6995e+05 | 1.8007e+05 | - 2.8484e+01 |
| 110Khz | 1.2147e+04 | -2.3816e+04 | 2.6735e+04 | - 5.2006e-01 |
| 1.01Mhz | 2.4590e+04 | -7.6780e+01 | 2.3968e+04 | - 5.5704e+01 |
| 1.51Mhz | 2.2190e+04 | -2.8932e+03 | 2.1370e+04 | - 7.2239e+01 |
| 2.01Mhz | 1.8830e+04 | -9.8949e+03 | 2.1269e+04 | - 3.3931 |

| **Beads24.9µm** | **SensorX2** | | | |
| --- | --- | --- | --- | --- |
| **Frequency** | **Real** | **Imag.** | **Mag.** | **Phase.** |
| 10Khz | 5.9735e+04 | -1.7003e+05 | 1.8008e+05 | - 3.5120e+01 |
| 110Khz | 1.1738e+04 | -2.4390e+04 | 2.6758e+04 | - 5.2111e+01 |
| 1.01Mhz | 2.4755e+04 | -3.6377e+01 | 2.4010e+04 | - 6.2739e+01 |
| 1.51Mhz | 2.2335e+04 | -2.9280e+03 | 2.1415e+04 | - 7.8162e+01 |
| 2.01Mhz | 1.8891e+04 | -9.8675e+03 | 2.1312e+04 | - 1.2500 |

| **Beads10.4µm** | **SensorX3** | | | |
| --- | --- | --- | --- | --- |
| **Frequency** | **Real** | **Imag.** | **Mag.** | **Phase.** |
| 10Khz | 5.9745e+04 | -1.6959e+05 | 1.7977e+05 | - 1.4986e+01 |
| 110Khz | 1.2128e+04 | -2.3419e+04 | 2.6361e+04 | - 8.2889 |
| 1.01Mhz | 2.5075e+04 | -4.4711e+02 | 2.3630e+04 | - 7.9495e+01 |
| 1.51Mhz | 2.1197e+04 | -3.1273e+03 | 2.1130e+04 | - 3.3870e+01 |
| 2.01Mhz | 1.9067e+04 | -9.2570e+03 | 2.1071e+04 | - 2.2216e+01 |

| **Beads24.9µm** | **SensorX3** | | | |
| --- | --- | --- | --- | --- |
| **Frequency** | **Real** | **Imag.** | **Mag.** | **Phase.** |
| 10Khz | 5.9546e+04 | -1.7014e+05 | 1.7997e+05 | - 4.8056e+01 |
| 110Khz | 1.2451e+04 | -2.3618e+04 | 2.6620e+04 | - 2.3474e+01 |
| 1.01Mhz | 2.3834e+04 | -9.2810e+02 | 2.3832e+04 | - 9.2120 |
| 1.51Mhz | 2.0908e+04 | -3.9113e+03 | 2.1268e+04 | - 3.3124 |
| 2.01Mhz | 1.8684e+04 | -1.0382e+04 | 2.1240e+04 | - 2.4510e+01 |

Table S4: The extracted numerical values for the impedance and phase of MCF7 at specific values of frequency using the different configurations

| **Viable PBS** | **Sensor1** | | | |
| --- | --- | --- | --- | --- |
| **Frequency** | **Real** | **Imag.** | **Mag.** | **Phase.** |
| 10Khz | 3.6420e+04 | -8.0759e+04 | 8.1960e+04 | -4.4277e+01 |
| 110Khz | 1.9221e+04 | -1.5748e+04 | 5.6404e+03 | -7.3349e+01 |
| 1.01Mhz | 1.6274e+04 | -1.2248e+04 | 2.0330e+04 | -1.9050e+01 |
| 1.51Mhz | 4.2427e+02 | -1.9972e+04 | 1.9700e+04 | -5.2158e+01 |
| 2.01Mhz | 1.3267e+04 | -1.4615e+04 | 1.9637e+04 | -3.0804e+01 |

| **Viable DMEM** | **Sensor1** | | | |
| --- | --- | --- | --- | --- |
| **Frequency** | **Real** | **Imag.** | **Mag.** | **Phase.** |
| 10Khz | 4.3145e+04 | -6.4334e+04 | 7.7438e+04 | -2.2050 |
| 110Khz | 2.0689e+04 | -2.5881e+03 | 1.8525e+04 | -5.4140e+01 |
| 1.01Mhz | 1.5297e+04 | -8.8464e+03 | 1.6817e+04 | -3.3622e+01 |
| 1.51Mhz | 2.8808e+03 | -1.7295e+04 | 1.6076e+04 | -4.3785e+01 |
| 2.01Mhz | 1.2462e+04 | -1.0690e+04 | 1.5962e+04 | -2.3549e+01 |

| **Nonviable PBS** | **Sensor1** | | | |
| --- | --- | --- | --- | --- |
| **Frequency** | **Real** | **Imag.** | **Mag.** | **Phase.** |
| 10Khz | 5.4004e+03 | -8.4347e+03 | 9.9872e+03 | -3.3295e+01 |
| 110Khz | 2.2367e+03 | -1.1827e+02 | 2.2378e+03 | -1.2110e+01 |
| 1.01Mhz | 1.6206e+03 | -1.2119e+03 | 2.0185e+03 | -2.0053e+01 |
| 1.51Mhz | 5.7741e+01 | -2.0043e+03 | 1.9608e+03 | -6.3127e+01 |
| 2.01Mhz | 1.3264e+03 | -1.4356e+03 | 1.9524e+03 | -1.3004e+01 |

| **Nonviable DMEM** | **Sensor1** | | | |
| --- | --- | --- | --- | --- |
| **Frequency** | **Real** | **Imag.** | **Mag.** | **Phase.** |
| 10Khz | 3.9081e+04 | -4.3303e+04 | 5.8226e+04 | -7.5886 |
| 110Khz | 3.8343e+03 | -8.7757e+03 | 8.5098e+03 | -5.1870e+01 |
| 1.01Mhz | 2.7087e+04 | -7.7608e+03 | 2.8039e+04 | -2.0128e+01 |
| 1.51Mhz | 7.4890e+03 | -1.1835e+03 | 6.1253e+03 | -6.6395e+01 |
| 2.01Mhz | 9.2149e+02 | -6.6634e+03 | 6.4333e+03 | -2.7400e+01 |

| **Viable PBS** | **X1** | | | |
| --- | --- | --- | --- | --- |
| **Frequency** | **Real** | **Imag.** | **Mag.** | **Phase.** |
| 10Khz | 5.2564e+04 | -1.6472e+05 | 1.7116e+05 | -3.4053e+01 |
| 110Khz | 4.6397e+03 | -1.8907e+04 | 1.7867e+04 | -2.7398e+01 |
| 1.01Mhz | 1.7990e+04 | -5.1376e+03 | 1.5629e+04 | -3.8286e+01 |
| 1.51Mhz | 1.4388e+04 | -1.3086e+03 | 1.3031e+04 | -2.4191e+01 |
| 2.01Mhz | 1.2076e+04 | -4.5160e+03 | 1.2672e+04 | -9.0653 |

| **Viable PBS** | **X2** | | | |
| --- | --- | --- | --- | --- |
| **Frequency** | **Real** | **Imag.** | **Mag.** | **Phase.** |
| 10Khz | 5.1983e+04 | -1.4355e+05 | 1.3154e+05 | -2.2325 |
| 110Khz | 1.1526e+04 | -1.9029e+04 | 2.0065e+04 | -1.6905e+01 |
| 1.01Mhz | 2.1908e+04 | -2.4924e+03 | 1.9330e+04 | -4.4909e+01 |
| 1.51Mhz | 2.0968e+04 | -7.0341e+02 | 1.6725e+04 | -6.3564e+01 |
| 2.01Mhz | 1.5521e+04 | -7.8335e+03 | 1.6569e+04 | -2.3833 |

| **Viable PBS** | **X3** | | | |
| --- | --- | --- | --- | --- |
| **Frequency** | **Real** | **Imag.** | **Mag.** | **Phase.** |
| 10Khz | 2.3915e+04 | -1.3727e+05 | 1.1910e+05 | -2.8162e+01 |
| 110Khz | 7.2251e+03 | -1.9246e+04 | 1.7941e+04 | -1.7938e+01 |
| 1.01Mhz | 2.0099e+04 | -1.7538e+03 | 1.7522e+04 | -2.7113e+01 |
| 1.51Mhz | 2.0953e+04 | -1.5962e+03 | 1.4902e+04 | -6.5707e+01 |
| 2.01Mhz | 1.5972e+04 | -5.9858e+03 | 1.4739e+04 | -1.9892e+01 |

| **Viable DMEM** | **X1** | | | |
| --- | --- | --- | --- | --- |
| **Frequency** | **Real** | **Imag.** | **Mag.** | **Phase.** |
| 10Khz | 5.3362e+04 | -1.3010e+05 | 1.3986e+05 | -9.6786 |
| 110Khz | 6.4180e+02 | -2.0840e+04 | 1.5113e+04 | -4.5682e+01 |
| 1.01Mhz | 1.8873e+03 | -5.8653e+03 | 2.7701e+03 | -1.1839e+01 |
| 1.51Mhz | 1.3331e+04 | -2.5011e+03 | 1.3563e+04 | -2.9901e-01 |
| 2.01Mhz | 1.9661e+04 | -1.0849e+03 | 1.3262e+04 | -5.9919e+01 |

| **Viable DMEM** | **X2** | | | |
| --- | --- | --- | --- | --- |
| **Frequency** | **Real** | **Imag.** | **Mag.** | **Phase.** |
| 10Khz | 3.7587e+03 | -1.6748e+05 | 1.2417e+05 | -6.7484e+01 |
| 110Khz | 1.7572e+03 | -1.8160e+04 | 1.5042e+04 | -3.2310e+01 |
| 1.01Mhz | 2.4553e+04 | -2.7834e+04 | 3.7515e+03 | -8.5715e+01 |
| 1.51Mhz | 1.4729e+04 | -8.0071e+02 | 1.3600e+04 | -2.2816e+01 |
| 2.01Mhz | 1.5592e+04 | -2.2139e+03 | 1.3256e+04 | -3.3908e+01 |

| **Viable DMEM** | **X3** | | | |
| --- | --- | --- | --- | --- |
| **Frequency** | **Real** | **Imag.** | **Mag.** | **Phase.** |
| 10Khz | 7.7189e+03 | -1.2484e+05 | 1.1118e+05 | -2.9584e+01 |
| 110Khz | 1.0290e+04 | -1.2191e+04 | 1.4997e+04 | -1.6826e+01 |
| 1.01Mhz | 8.0301e+03 | -1.7071e+04 | 1.6026e+02 | -4.3865e+01 |
| 1.51Mhz | 1.3348e+04 | -2.2449e+03 | 1.3527e+04 | -1.8782 |
| 2.01Mhz | 1.4661e+04 | -2.7308e+03 | 1.3204e+04 | -2.7434e+01 |

| **Nonviable PBS** | **X1** | | | |
| --- | --- | --- | --- | --- |
| **Frequency** | **Real** | **Imag.** | **Mag.** | **Phase.** |
| 10Khz | 6.0012e+03 | -1.7042e+04 | 1.8050e+04 | -6.3354e+01 |
| 110Khz | 1.2325e+03 | -2.4219e+03 | 2.7174e+03 | -2.8179 |
| 1.01Mhz | 2.5127e+03 | -8.2608e+01 | 2.4883e+03 | -6.4245e+01 |
| 1.51Mhz | 1.9108e+03 | -4.8017e+02 | 1.3121e+03 | -5.9473e+01 |
| 2.01Mhz | 1.9500e+03 | -1.0243e+03 | 2.2021e+03 | -7.4931 |

| **Nonviable PBS** | **X2** | | | |
| --- | --- | --- | --- | --- |
| **Frequency** | **Real** | **Imag.** | **Mag.** | **Phase.** |
| 10Khz | 5.7509e+03 | -1.4012e+04 | 1.5036e+04 | -1.4152e+01 |
| 110Khz | 8.1493e+02 | -1.8480e+03 | 2.0079e+03 | -8.7215 |
| 1.01Mhz | 2.5222e+03 | -1.1055e+03 | 1.3037e+03 | -8.6814e+01 |
| 1.51Mhz | 1.8324e+03 | -7.8837e+02 | 9.9516e+02 | -6.0814e+01 |
| 2.01Mhz | 9.1559e+02 | -3.1801e+02 | 9.5140e+02 | -6.1538 |

| **Nonviable PBS** | **X3** | | | |
| --- | --- | --- | --- | --- |
| **Frequency** | **Real** | **Imag.** | **Mag.** | **Phase.** |
| 10Khz | 3.5101e+03 | -1.2852e+04 | 1.3187e+04 | -1.1546e+01 |
| 110Khz | 1.0283e+03 | -1.7408e+03 | 2.0073e+03 | -9.6786 |
| 1.01Mhz | 2.0571e+03 | -2.4712e+02 | 1.9319e+03 | -3.5307e+01 |
| 1.51Mhz | 2.0438e+03 | -1.4988e+02 | 1.6678e+03 | -6.0780e+01 |
| 2.01Mhz | 1.6311e+03 | -5.6107e+02 | 1.6501e+03 | -2.4591e+01 |

| **Nonviable DMEM** | **X1** | | | |
| --- | --- | --- | --- | --- |
| **Frequency** | **Real** | **Imag.** | **Mag.** | **Phase.** |
| 10Khz | 4.3336e+03 | -1.3597e+04 | 1.4237e+04 | -6.7544 |
| 110Khz | 1.1031e+02 | -2.4220e+03 | 1.6133e+03 | -6.1392e+01 |
| 1.01Mhz | 1.3938e+03 | -2.3333e+02 | 1.3420e+03 | -1.4651e+01 |
| 1.51Mhz | 1.4327e+03 | -2.6756e+02 | 1.4574e+03 | -4.4583e-01 |
| 2.01Mhz | 1.3688e+03 | -4.8493e+02 | 1.4138e+03 | -1.3738e+01 |

| **Nonviable DMEM** | **X2** | | | |
| --- | --- | --- | --- | --- |
| **Frequency** | **Real** | **Imag.** | **Mag.** | **Phase.** |
| 10Khz | 2.2885e+03 | -1.6175e+04 | 1.4237e+04 | -5.7410e+01 |
| 110Khz | 6.1203e+02 | -1.5196e+03 | 1.6225e+03 | -7.3528 |
| 1.01Mhz | 1.7497e+03 | -6.8661e+02 | 1.4079e+03 | -4.3275e+01 |
| 1.51Mhz | 1.6401e+03 | -1.4907e+02 | 1.4472e+03 | -3.3244e+01 |
| 2.01Mhz | 1.3711e+03 | -4.8076e+02 | 1.4127e+03 | -1.4050e+01 |

| **Nonviable DMEM** | **X3** | | | |
| --- | --- | --- | --- | --- |
| **Frequency** | **Real** | **Imag.** | **Mag.** | **Phase.** |
| 10Khz | 2.4579e+03 | -2.4579e+03 | 1.4134e+04 | -4.5423e+01 |
| 110Khz | 2.7501e+02 | -2.7501e+02 | 1.6286e+03 | -3.3087e+01 |
| 1.01Mhz | 2.3321e+03 | -2.3321e+03 | 4.2688e+02 | -8.1793e+01 |
| 1.51Mhz | 1.3938e+03 | -1.3938e+03 | 1.4313e+03 | -6.0286 |
| 2.01Mhz | 2.0040e+03 | -2.0040e+03 | 1.3977e+03 | -6.2212e+01 |

Table S5. Differentiation indexes for electrical impedances for microbeads concerning measured frequency

| **Sensor1**  DIFFERENTIATION INDEXES | | | | |
| --- | --- | --- | --- | --- |
| **Frequency** | **Real** | **Img.** | **Mag.** | **Phase.** |
| 10Khz | -1.90357 | 2.716777 | -0.71302 | -4.16418 |
| 110Khz | -1.34785 | -0.75935 | -0.57687 | -0.85141 |
| 1.01Mhz | 2.33731 | -0.9159 | 2.319164 | -0.25039 |
| 1.51Mhz | 3.172783 | -1.12207 | 2.344138 | -9.10099 |
| 2.01Mhz | 2.510412 | -1.05632 | 2.359164 | -3.46615 |

| **SensorX1**  Differentiation indexes | | | | |
| --- | --- | --- | --- | --- |
| **Frequency** | **Real** | **Img.** | **Mag.** | **Phase.** |
| 10Khz | -6.2431 | 3.468458 | -2.45476 | 5.744924 |
| 110Khz | -10.4813 | 3.976372 | -3.98931 | 6.929374 |
| 1.01Mhz | 2.532733 | -13.1105 | 3.458596 | 2.6384 |
| 1.51Mhz | 3.320685 | -2.78954 | 3.71778 | 34.98886 |
| 2.01Mhz | 3.614589 | -154.132 | 3.892843 | -9.83937 |

Table S6. Differentiation indexes for electrical impedances for MCF7 in PBS concerning measured frequency

| **Sensor1**  Differentiation indexes | | | | |
| --- | --- | --- | --- | --- |
| **Frequency** | **Real** | **Img.** | **Mag.** | **Phase.** |
| 10Khz | 0.748128 | 1.53802 | 0.395834 | 4.294424 |
| 110Khz | 0.101338 | 2.340782 | -0.87921 | -7.52639 |
| 1.01Mhz | 0.362165 | 2.514922 | -0.75733 | 2.444613 |
| 1.51Mhz | 0.791604 | 1.634236 | -0.65251 | 6.701561 |
| 2.01Mhz | 0.699252 | 2.396187 | -0.78006 | 11.63678 |

| **SensorX1**  Differentiation indexes | | | | |
| --- | --- | --- | --- | --- |
| **Frequency** | **Real** | **Img.** | **Mag.** | **Phase.** |
| 10Khz | 4.612519 | 4.612519 | 7.626409 | -0.34117 |
| 110Khz | 5.182198 | 5.182198 | 1.993136 | 0.18393 |
| 1.01Mhz | 2.682978 | 2.682978 | 1.335109 | 3.015128 |
| 1.51Mhz | 3.705383 | 3.705383 | 4.473086 | -1.97637 |
| 2.01Mhz | 5.555673 | 5.555673 | 4.185826 | -8.82602 |

Table S7. Differentiation indexes for electrical impedances for MCF7 in DMEM concerning measured frequency

| **Sensor1**  Differentiation indexes | | | | |
| --- | --- | --- | --- | --- |
| **Frequency** | **Real** | **Img.** | **Mag.** | **Phase.** |
| 10Khz | -0.89972 | -0.71451 | -10.2003 | 1.886161 |
| 110Khz | -1.08875 | 2.343574 | -0.24068 | 2.221563 |
| 1.01Mhz | -0.75138 | 2.261277 | -0.25643 | -2.05394 |
| 1.51Mhz | -0.51564 | 2.897665 | -0.22573 | 1.039624 |
| 2.01Mhz | -0.38681 | 2.480067 | -0.22798 | 0.042772 |

| **SensorX1**  DIFFERENTIATION INDEXES | | | | |
| --- | --- | --- | --- | --- |
| **Frequency** | **Real** | **Img.** | **Mag.** | **Phase.** |
| 10Khz | 3.157324 | -1.25723 | 2.990151 | -4.8373 |
| 110Khz | -1.11087 | 5.905306 | -1.56902 | 1.741301 |
| 1.01Mhz | -3.40793 | 3.665924 | -1.42613 | 9.4784 |
| 1.51Mhz | -15.2304 | 6.493103 | -4.84348 | -1.89685 |
| 2.01Mhz | 5.045971 | 3.201582 | -1.67174 | -0.61199 |

The results in the table demonstrate that the system can accurately distinguish between cells based on their electrical impedance properties, as indicated by the Differentiation Index (D) values at each frequency. The D values show that the system can effectively differentiate between the cells in terms of the impedance's real, imaginary, magnitude, and phase components. These results indicate that the system has robust capabilities for detecting differences between cells and can do so even at high frequencies, such as 2.01 MHz. The system's sensitivity in detecting these differences is also evident from the results. These results demonstrate the system's effectiveness in differentiating between cells based on their electrical impedance properties.

**A**

Impedance |Z| Ω

**B**

Impedance |Z| Ω

Fig. S1. Peak Value of Electrical impedance responses of Microbeads as a function of frequency for magnitude part at the different frequency Fig.S1A) Sensor 1. Fig.S1B) Modified electrodes with the same number X1. The vertical bars represent the error defined by maximum and minimum values.

Impedance |Z| Ω

Impedance |Z| Ω

Fig. S2. Peak Value of Electrical impedance responses of MCF7 as a function of frequency for magnitude part at the different frequency Fig.S2A) Sensor 1 vs. PBS as a buffer solution. Fig.S2B) Sensor X1 vs. PBS as a buffer solution. Fig.S2C) using Sensor 1 when DMEM is the buffer solution. Fig.S2D) Sensor x1 for DMEM as the buffer solution. The vertical bars represent the error defined by maximum and minimum values.
